# Extracellular Vesicles from *Capparis spinosa* Modulate Epithelial-to-Mesenchymal Transition in Huh7 Hepatocellular Carcinoma Cells

**DOI:** 10.3390/nano16070394

**Published:** 2026-03-25

**Authors:** Agnese Bertoldi, Eleonora Calzoni, Gaia Cusumano, Husam B. R. Alabed, Roberto Maria Pellegrino, Carla Emiliani, Lorena Urbanelli

**Affiliations:** 1 Department of Chemistry, Biology and Biotechnology, University of Perugia, 06100 Perugia, Italy; agnese.bertoldi@dottorandi.unipg.it (A.B.); gaia.cusumano@dottorandi.unipg.it (G.C.); husambr.alabed@unipg.it (H.B.R.A.); roberto.pellegrino@unipg.it (R.M.P.); carla.emiliani@unipg.it (C.E.); lorena.urbanelli@unipg.it (L.U.); 2 Centro di Eccellenza Materiali Innovativi Nanostrutturati (CEMIN), University of Perugia, Via del Giochetto, 06123 Perugia, Italy

**Keywords:** epithelial–mesenchymal transition (EMT), hepatocellular carcinoma, plant-derived extracellular vesicles (PDEVs), *Capparis spinosa*, nanocarriers, lipidomics, metabolomics

## Abstract

**Background:** Epithelial-to-mesenchymal transition (EMT) is a cellular reprogramming process characterized by coordinated changes in signaling, membrane organization and metabolism. In a previously established and deeply characterized Huh7 EMT model, it was demonstrated that TGF-β stimulation induces a reproducible shift toward a mesenchymal state accompanied by lipidomic and metabolic remodeling. Building on this framework, the present study evaluates whether extracellular vesicles (EVs)-enriched fractions derived from *Capparis spinosa* can modulate these EMT-associated alterations. **Methods:** After detailed physicochemical, molecular, lipidomic and metabolomic characterization, *C. spinosa* EVs were applied to EMT-induced Huh7 cells. The vesicles were efficiently internalized and, while not inducing a complete epithelial reversion, they attenuated mesenchymal features, indicating a modulatory rather than inhibitory action. **Results:** Lipidomic profiling showed a partial correction of TGF-β-induced changes including diacylglycerols, phosphoinositides and triglycerides, suggesting interference with lipid signaling and membrane turnover. Metabolomic data further points to reduced mitochondrial and fatty acid oxidation stress, reflected in the re-equilibration of carnitine and acylcarnitine species. **Conclusions:** Together, these findings indicate that *C. spinosa* EVs are able to attenuate EMT-associated metabolic and membrane remodeling, positioning them as promising modulators of tumor cell plasticity.

## 1. Introduction

The epithelial–mesenchymal transition (EMT) is a dynamic and reversible biological program in which epithelial cells, characterized by tight junctions, apico–basal polarity and adhesion to the basement membrane, undergo a phenotypic conversion toward a mesenchymal state [1,2,3,4]. This process involves disassembly of intercellular junctions, loss of epithelial markers such as E-cadherin, reorganization of the actin cytoskeleton, and acquisition of a spindle-shaped morphology associated with increased motility and invasiveness. Although traditionally described as a binary switch, EMT occurs along a continuum of intermediate phenotypes referred to as partial EMT (pEMT), where epithelial and mesenchymal traits coexist, conferring high cellular plasticity and context-dependent adaptability [5,6]. EMT contributes to development and tissue repair, but also to pathological conditions including fibrosis, tumor progression and metastasis formation [1,7,8].

Hepatocellular carcinoma (HCC), one of the most lethal malignancies worldwide, frequently shows EMT peculiarities to enhance invasiveness, vascular dissemination and resistance to therapy. Chronic liver injury provides sustained exposure to cytokines and growth factors, such as TGF-β, HGF, IL-6 and hypoxia, which compromise epithelial identity and favor the emergence of mesenchymal traits. In particular, loss of E-cadherin and induction of Vimentin or N-cadherin correlate with vascular invasion and poor prognosis in HCC patients [9,10]. Beyond transcriptional reprogramming, EMT in HCC is associated with marked metabolic changes, especially in lipid metabolism. TGF-β-induced EMT promotes increased lipid turnover, accumulation of free fatty acids and upregulation of fatty acid transporters such as FABPs and SLC27 family members [11,12,13]. HCC cells with marked EMT traits import exogenous fatty acids and direct them toward fatty acid oxidation and oxidative phosphorylation, a metabolic configuration that sustains migration and survival under stress [11,14,15]. Pharmacological or genetic inhibition of lipid uptake can attenuate EMT phenotypes, although the metabolic response varies among HCC models, reflecting different degrees of EMT commitment [11,12,16]. Within this context, the Huh7 cell line represents a suitable model to examine EMT-associated metabolic remodeling. In a previous study [17], we established and characterized a robust TGF-β-induced EMT model in Huh7 cells, documenting transcriptional, morphological, lipidomic and metabolomic changes consistent with a shift toward mesenchymal identity. This existing framework provides the baseline for investigating factors capable of modulating EMT-linked alterations.

Plant-derived extracellular vesicles (PDEVs) are nanosized lipid bilayer particles naturally released by plant cells and detectable in juices, apoplastic fluids and culture. Ranging from 50 to 500 nm, PDEVs protect and transport lipids, metabolites, nucleic acids and secondary compounds, including polyphenols, carotenoids and phytosterols, which possess antioxidant, anti-inflammatory and anticancer activities [18]. Their structural similarity to mammalian EVs, combined with high biocompatibility and stability, makes them promising candidates as natural nanocarriers. *Capparis spinosa*, a Mediterranean medicinal plant rich in flavonoids (rutin, quercetin, kaempferol derivatives), alkaloids, glucosinolates, phytosterols and terpenoids [19,20,21], exhibits antioxidant, anti-inflammatory, antifibrotic, metabolic and hepatoprotective activities [22,23,24,25,26,27,28]. For example, in experimental models of tert-butyl hydroperoxide-induced acute liver injury, hydroalcoholic extracts of *C. spinosa* have been shown to significantly reduce serum liver enzyme levels and lipid peroxidation while restoring antioxidant defenses such as glutathione (GSH), superoxide dismutase (SOD), and catalase (CAT), effects largely attributed to its high phenolic and quercetin content [29]. Many of its effects converge on pathways central to EMT regulation, including oxidative stress responses, extracellular matrix remodeling and TGF-β signaling [22,23,24,25]. This mechanistic overlap suggests that vesicles derived from *C. spinosa* may influence EMT-associated cellular states.

Based on this rationale, the present study investigates the capacity of *C. spinosa* extracellular vesicle-enriched fraction to modulate transcriptional, metabolic and lipidomic alterations previously defined in our TGF-β-induced EMT model in Huh7 cells.

## 2. Materials and Methods

### 2.1. Isolation Procedure of PDEVs from C. spinosa Fruit

The fruit of *C. spinosa* was purchased from a local herbal shop. For extracellular vesicle isolation, 5 gr of freeze-dried biomass were weighed and rehydrated in 20 mL of VIB extraction buffer (20 mM MES, 2 mM CaCl_2_, 0.1 M NaCl, pH 6.0). Through the application of positive and negative pressures, the saline buffer was infiltrated into the tissue. Nanovesicles were isolated by differential ultracentrifugation (dUC) of the Apoplastic Fluid (AF) as described by Chiaradia et al. [30]. An initial low-speed centrifugation at 700× *g* for 20 min at 4 °C was performed to obtain the AF included in the tissue. The supernatant was then filtered through a 0.45 µm membrane and subjected to sequential centrifugation steps: first at 10,000× *g* for 60 min, followed by 100,000× *g* for another 60 min. The final pellet, enriched in nanovesicles, was resuspended in different solvents according to the intended downstream applications: 1000 µL of PBS for NTA assays, 200 µL of PBS supplemented with 10% DMSO for cell-based studies.

### 2.2. Characterization of PDEVs: Nanoparticle Tracking Analysis (NTA) and Scanning Electron Microscopy (SEM)

The particle size distribution and concentration of caper nanovesicles were determined using a NanoSight NS300 system (Malvern Panalytical, Malvern, UK) for nanoparticle tracking analysis (NTA). Prior to analysis, vesicle preparations were diluted in sterile PBS filtered through a 0.45 µm membrane to obtain the recommended particle range for the instrument [31]. Three independent biological preparations were analyzed. Each sample underwent five consecutive readings, with measurements repeated in two independent experimental runs. For Scanning Electron Microscopy (SEM), nanovesicles were resuspended in 50 µL of 0.22 µm filtered PBS. Protein content was assessed via the Bradford assay [32], performed according to the manufacturer’s guidelines. An aliquot corresponding to 2 µg of total protein was mixed with 2 mL of 2.5% glutaraldehyde (*v*/*v* in 1× PBS) and incubated at room temperature for 15 min to allow fixation. Following fixation, the mixture was diluted with 15 mL of double-distilled, 0.22 µm filtered water and concentrated using Vivaspin centrifugal filters (MWCO: 300 kDa). Samples were centrifuged at 3000× *g* for 3 min, and the flow-through was discarded. The washing procedure was repeated twice with 10 mL of ultrapure water under the same centrifugation conditions. For SEM mounting, vesicle suspensions were further diluted at 1:500 and 1:1000 ratios, and 20 µL of each dilution was placed onto 12 mm glass coverslips. After allowing adhesion, the samples were fixed, sputter-coated with a thin conductive layer, and subsequently imaged.

### 2.3. Immunoblotting and Proteinase K Protection Assay

Extracellular vesicle-enriched fraction and total plant extracts were lysed in Laemmli sample buffer (final composition: 62.5 mM Tris-HCl pH 6.8, 2% SDS, 10% glycerol, 0.01% bromophenol blue) supplemented with 125 mM dithiothreitol (DTT). Protein concentration was determined using the Bradford assay (Bio-Rad, Hercules, CA, USA). Equal amounts of protein (10 μg per lane) were loaded onto 10% SDS-polyacrylamide gels and separated by SDS-PAGE under reducing conditions. Proteins were transferred onto polyvinylidene difluoride membranes using the Trans-Blot Turbo Transfer System (Bio-Rad, Hercules, CA, USA). Membranes were blocked in 5% non-fat dry milk in TBS-T (Tris-buffered saline, 0.1% Tween-20) for 1 h at room temperature and incubated overnight at 4 °C with primary antibodies against TET8 (PhytoAB, San Jose, CA, USA) and Calnexin (Santa Cruz Biotechnology, Santa Cruz, CA, USA). After washing, membranes were incubated with appropriate HRP-conjugated secondary antibodies (Cell Signaling Technology, Danvers, MA, USA) for 1 h at room temperature. Protein bands were detected using enhanced chemiluminescence (ECL, GE Healthcare, Chicago, IL, USA) and visualized with a chemiluminescence imaging system. Densitometric analysis was performed using ImageJ 1.53 software (NIH, Bethesda, MD, USA).

To assess membrane protection of vesicle-associated proteins, EV preparations were subjected to protease digestion in the presence or absence of detergent. Briefly, EV samples (10 μg total protein) were incubated with proteinase K (100 μg/mL final concentration) for 30 min at 37 °C. Where indicated, 0.1% Triton X-100 was added to permeabilize the vesicular membrane. Reactions were stopped by adding phenylmethylsulfonyl fluoride (PMSF, 5 mM final concentration) and immediately mixed with Laemmli sample buffer followed by heat denaturation at 95 °C for 5 min. Samples were then analyzed by SDS-PAGE and immunoblotting as described above.

### 2.4. LC–QTOF Characterization of Lipid and Polyphenol Content in C. spinosa PDEVs

PDEVs were isolated by differential ultracentrifugation and stored at −80 °C until analysis. For lipidomics, EV suspensions (50 µL) were extracted using a modified MMC protocol [33] by mixing with 1 mL of methanol/methyl tert-butyl ether/chloroform (1:1:1, *v*/*v*/*v*). After vortexing, the samples were centrifuged at 13,000× *g* for 10 min at 4 °C. The organic phase was collected, evaporated under a nitrogen stream at 60 °C, and reconstituted in methanol/toluene (9:1, *v*/*v*) for LC-MS analysis. For the analysis of polyphenols, 40 µL of 70% methanol containing 3% formic acid were added to the PDEVs, which were vortexed, centrifuged at 10,000× *g* for 10 min at 4 °C, and the supernatant was transferred to glass vials for subsequent LC–QTOF analysis.

All samples were analyzed on an Agilent 1260 Infinity II UHPLC system coupled to an Agilent 6530 quadrupole time-of-flight mass spectrometer equipped with a JetStream electrospray ionization source (Agilent Technologies, Santa Clara, CA, USA). Mass spectrometry was performed in both positive and negative ionization modes over a mass range of *m*/*z* 40–1700, with the following parameters: capillary voltage 3500 V, drying gas temperature 250 °C, sheath gas temperature 300 °C, nebulizer pressure 35 psi, sheath gas flow 12 L min^−1^, and collision energy 30 V for data-dependent MS/MS acquisition.

Chromatographic separation of lipids was achieved on a Waters Acquity C18 UPLC column (Waters Corporation, Milford, MA, USA) (100 × 2.1 mm, 1.7 µm) maintained at 60 °C with a flow rate of 0.30 mL min^−1^, using a binary solvent system consisting of acetonitrile/water (60:40) with 10 mM ammonium acetate (solvent A) and isopropanol/acetonitrile (90:10) with 10 mM ammonium acetate (solvent B). Polyphenols were separated on a Waters Acquity BEH C18 column (150 × 2.1 mm, 1.7 µm) maintained at 25–30 °C with a flow rate of 0.35 mL min^−1^. The mobile phases consisted of water (A) and acetonitrile (B), both containing 0.2% formic acid, with a gradient starting at 5% B, increasing to 45% over 15 min, then rising to 95% between 15 and 18 min, held for 2 min, and re-equilibrated to 5% B at 20.1 min, for a total run of 23 min.

Raw MS data were processed using Agilent MassHunter Qualitative Analysis and MS-DIAL software (versions 4.48–4.9) for peak detection, alignment, integration, and annotation. Polar metabolites and polyphenols were identified by matching against the NIST2020 database, while lipids were annotated using the LSG database and further analyzed with the LipidONE 2.3 platform [34]. In the absence of internal standards for the polyphenol assay, relative abundances were expressed as the percentage contribution to the total phenolic content (TPC) measured spectrophotometrically.

### 2.5. EMT Induction in Hepatocellular Carcinoma Cells and PDEVs Treatment

Human HCC cell line Huh7 was purchased from ATCC (Manassas, VA, USA) and maintained at 37 °C in a humidified incubator with 5% CO_2_, using DMEM supplemented with 10% heat-inactivated FBS and antibiotics (100 U/mL penicillin and 100 U/mL streptomycin). For the induction of the epithelial-to-mesenchymal transition process, cells were plated 24 h before treatment, reaching a confluence of 70%. Cells were cultured in standard medium supplemented with recombinant human TGF-β1 protein (10 ng/mL, Cell Signaling Technology, Beverly, MA, USA), PDEVs, or a combination of both, in order to generate four experimental conditions: untreated control, TGF-β1 alone, PDEVs alone, and PDEVs plus TGF-β1. A protein concentration of 0.6 µg was used as the standard treatment dose for extracellular vesicles derived from *C. spinosa*. Culture media were refreshed daily, and treatments were maintained for 48 h. Cell viability was assessed using trypan blue exclusion and quantified with an automated cell counter (Invitrogen™ Countess™, Thermo Fisher Scientific, Waltham, MA, USA) and MTT assay for the evaluation of TGF-β1 cytotoxicity, as reported by Calzoni et al. [31].

### 2.6. C. spinosa PDEVs Uptake by Fluorescence Microscopy

The uptake of PDEVs by hepatocyte was evaluated by assessing the intracellular localization of EVs labeled with DiL (1,1′-Dioctadecyl-3,3,3′,3′-Tetramethylindocarbocyanine Perchlorate) by fluorescence microscopy. A concentration of 50 µM DiL was added to the supernatant obtained after centrifugation at 10,000× *g* and to PBS used as control. Following a 30 min incubation at room temperature, the sample was processed through the same centrifugation and filtration steps as previously described for the isolation of PDEVs. For fluorescence microscopy analysis, Huh7 cells (5 × 10^4^) were incubated with DiL-labeled NVs for 1 h. After incubation, cells were fixed with 4% paraformaldehyde for 20 min. F-actin was stained using fluorescein isothiocyanate (FITC)-conjugated phalloidin for 30 min. The samples containing staining nuclei with VECTASHIELD Vibrance^®^ Antifade Mounting Medium containing DAPI were then mounted and image acquisition was performed using a fluorescence microscope (Eclipse TE2000-S, Nikon, Tokyo, Japan) equipped with the F-View II FireWire camera (Olympus Soft Imaging Solutions, Münster, Germany) and through the use of CellF Imaging Software (Olympus Soft Imaging Solutions).

### 2.7. Gene Expression Analysis Through Quantitative Real-Time Polymerase Chain Reaction (qRT-PCR)

qRT-PCR was performed to determine the expression levels of EMT-related genes. Total RNA was isolated and extracted from cells using TRIzol RNA Isolation Reagents (Invitrogen, Karlsruhe, Germany). Then 1 μg of RNA was reverse-transcribed into cDNA by using random hexamers and SuperScript II Reverse Transcriptase (Invitrogen) according to the manufacturer’s protocol. Quantitative real-time PCR (RT-PCR) was performed with the primers already used in previous study [17]. Products were detected with SYBR Green Master Mix (Applied Biosystems, Foster City, CA, USA) using a StepOnePlus thermocycler (Applied Biosystems, Foster City, CA, USA). cDNA abundance was normalized against GAPDH genes. Relative quantification was obtained using the 2^−∆∆Ct^ method.

### 2.8. Immunoblotting

Cells were collected by centrifugation, and the resulting pellets were lysed in RIPA buffer (50 mM Tris-HCl pH 8, 150 mM NaCl, 1% (*v*/*v*) Igepal CA-630, 0.1% (*w*/*v*) SDS, 0.5% (*w*/*v*) sodium deoxycholate) supplemented with a protease inhibitor cocktail (Merck Life Sciences, Darmstadt, Germany). After incubation, cell debris was eliminated by centrifugation at 13,000× *g* for 10 min at 4 °C. Aliquots of cell lysates (30 μg proteins) were mixed with a 5× sample buffer (1 M Tris–HCl pH 6.8, 5% SDS, 6% glycerol, 0.01% bromophenol blue) containing 125 mM DTT, and then heated at 95 °C for 5 min to denature the proteins. Samples were then loaded onto a 10% polyacrylamide gel for SDS-PAGE and transferred onto PVDF membranes using the Trans-Blot Turbo Transfer System (Bio-Rad, Hercules, CA, USA). After blocking, membranes were incubated overnight with the following primary antibodies: Vimentin mAb and N-cadherin mAb (Cell Signaling Technology (Beverly, MA, USA)) and β-actin mAb (Sigma-Aldrich, St. Louis, MO, USA). Detection was performed using HRP-conjugated secondary antibodies (Cell Signaling Technology) according to the manufacturer’s instructions. Immunoreactive bands were visualized via enhanced chemiluminescence (ECL, GE Biosciences, Chicago, IL, USA), and densitometric quantification was conducted using ImageJ software (NIH, Bethesda, MD, USA).

### 2.9. Seahorse Glycolytic Activity Analysis

The glycolytic activity of all four combinations of treatment cells was analyzed by an Agilent Seahorse XFp Extracellular Flux Analyzer (Agilent Technologies, Santa Clara, CA, USA) using a Glycolytic Rate assay kit. Cells (2.5 × 10^4^/well) were seeded in XFp cell culture microplates, and after 24 h the medium was replaced with a fresh medium with different a combination of treatment; replacement with fresh medium was performed daily. Upon reaching 48 h of exposure to the cytokine and PDEVs, the medium was replaced with phenol red-free XF DMEM medium supplemented with 10 mM glucose, 2 mM sodium pyruvate, and 2 mM glutamine. To block the electron transport chain and prevent the production of protons derived from CO_2_, mitochondrial respiration was inhibited by adding Rotenone and Antimycin A (Rot/AA), while for the suppression of glycolytic activity, 2-deoxy-D-glucose (2-DG), a glucose analog, was administered to competitively inhibit hexokinase, the key enzyme initiating glycolysis. The proton efflux rate (PER) is used to show the effects of the induction of the transition process on the glycolytic activity resulting from cellular metabolism.

### 2.10. Lipid and Polar Metabolites Analysis of EMT Model Treated with PDEVs by Liquid Chromatography-Tandem Mass Spectrometry (LC–MS/MS)

For both polar and apolar (lipids) metabolite analysis, aliquots of cells (1 × 10^6^ cells) were prepared for the metabolite extraction. The extraction followed a biphasic protocol reported by Cajka et al. [35], with slight modifications. Each cell pellet was first mixed with 275 µL of methanol containing the Lipidomix SPLASH internal standard and vortexed for 30 s. Then, 750 uL of methyl tert-butyl ether (MTBE) was added, and the samples were vortexed again, followed by 20 min of shaking at 1500 rpm on a T-Shaker (Euroclone, Pero, Italy). Next, 188 uL of water was added for the samples. The samples were centrifuged at 16,000× *g* for 10 min at 4 °C to achieve phase separation.

The upper (organic) phase was recovered and dried under a nitrogen steam at 60 °C. The dry extract was reconstituted in 100 µL of MeOH/Toluene (9:1, *v*/*v*) for the lipidomics analysis. The same process was done for the lower (aqueous) phase, which was carefully collected into a glass vial and evaporated under nitrogen stream at 60 °C. The resulting dry extract was reconstituted in 100 µL of a 4:1 acetonitrile/water solution and placed in the autosampler for polar metabolite analysis. Chromatographic separation and data acquisition was performed using an UHPLC-QTOF system (Agilent 1260 Infinity II liquid chromatograph coupled with an Agilent 6530 Accurate-Mass Q-TOF mass spectrometer equipped with a JetStream ESI source). The LC-MS settings for both lipid and polar metabolite analysis followed the protocol described by Alabed et al. [36].

### 2.11. Wound Healing Assay

Huh7 cells were seeded in 6-well plates and grown to approximately 90–100% confluence. A linear scratch was created using a sterile 200 µL pipette tip. Detached cells were removed by washing with PBS, and fresh medium containing the appropriate treatments (CTRL, C. spinosa EVs, TGF-β, or TGF-β + EVs) was added. Images were acquired at 0, 24, and 48 h using an inverted phase-contrast microscope. Wound width was measured at defined reference points using ImageJ software. Migration was quantified as the percentage of wound closure relative to time 0 according to the formula:

% wound closure = [(initial wound width − wound width at time X)/initial wound width] × 100.

## 3. Results

### 3.1. C. spinosa PDEVs Characterization

#### 3.1.1. SEM, NTA Characterization and Immunoblotting Analysis

**Figure 1:**
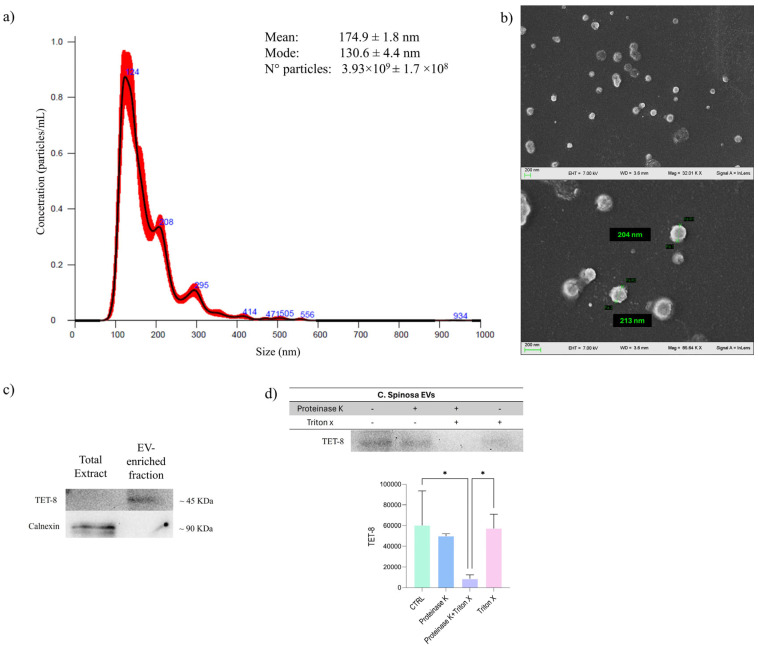
NTA (**a**) and SEM images (**b**) of *C. spinosa* EVs. Data are expressed as mean ± SD (n = 5). Western blot analysis of EV-associated marker TET8 (**c**) and proteinase K protection assay performed to assess membrane protection of EV-associated proteins (**d**). The images shown are representative of independent experiments. Full, uncropped blots are available in Supplementary Figure S1. Data are expressed as mean ± SD (n = 3); statistical significance is indicated as * *p* < 0.05.

To evaluate the outcome of the differential centrifugation steps performed on *Capparis spinosa*, size distribution and morphological characterization was carried out using Nanoparticle tracking analysis (NTA) and Scanning Electron Microscopy (SEM) (Figure 1).

The NTA of *C. spinosa* EV-enriched fraction showed an average diameter of 174.9 ± 1.8 nm, with a mode of 130.6 ± 4.4 nm (Figure 1a). The size distribution remained within a well-defined range, with no signal detected above 500 nm, indicating a fairly homogeneous particle population. The consistency between the mean and mode values thus provides an initial quantitative validation of both the efficiency and quality of the isolation process. In addition, NTA allowed us to estimate the particle concentration, which reached 3.93 × 10^9^ ± 1.7 × 10^8^ particles, confirming that the isolation protocol yields a substantial amount of EVs from the plant source. To further confirm the membranous nature of the detected nanoparticles, NTA was repeated after treatment with Triton X-100. As shown in Figure S2, detergent exposure resulted in a dramatic reduction in particle concentration and the disappearance of the main vesicular peak, with only a minor residual signal corresponding to small non-vesicular particles. This detergent-sensitive behavior supports the presence of intact lipid bilayer-delimited vesicles in the untreated preparation.

To investigate vesicle morphology, Scanning Electron Microscopy was performed (Figure 1b). SEM images of EVs from *C. spinosa* showed spherical vesicles with diameters ranging between 200 and 215 nm, characterized by a slightly rough surface, which is typical of plant-derived EVs [31].

Western blot analysis revealed enrichment of TET8, a well-established plant extracellular vesicle marker, in the EV-enriched fraction compared to the total extract (Figure 1c). Instead, Calnexin, an endoplasmic reticulum marker, was detected in the total extract but was absent in the EV fraction, indicating minimal contamination from intracellular membrane compartments. To further validate the vesicular nature of the preparation, a proteinase K protection assay was performed. Treatment with proteinase K alone resulted in partial preservation of TET8 signal (Figure 1d), consistent with protection of intravesicular domains by an intact lipid bilayer. In contrast, combined treatment with proteinase K and Triton X-100 led to a marked reduction in TET8 signal, indicating a loss of membrane integrity and proteolytic accessibility. This detergent-dependent protease sensitivity confirms that TET8 is associated with membrane-protected vesicular structures.

#### 3.1.2. Polyphenolic and Lipid Profile of *Capparis spinosa* EVs

The phenolic composition of *C. spinosa* EV-enriched fraction was characterized through untargeted mass spectrometry. Quantitative normalization was carried out against the TPC, determined through the Folin–Ciocalteu assay, thus allowing both the qualitative identification and relative quantification of the metabolites detected (Table 1) [37].

The analysis revealed a heterogeneous spectrum of secondary metabolites, including flavonoids (flavonols, isoflavones, proanthocyanidins), galloyl/coumaroyl derivatives, simple phenolic acids, and triterpenoids. Among these, several compounds were particularly abundant and are known to exert significant biological functions.

Rutin, or Quercetin-3-O-rutinoside, was by far the most abundant metabolite. This glycosylated flavonol is widely recognized for its strong antioxidant and anti-inflammatory activities, but more importantly, it is consistently reported as a potent modulator of epithelial–mesenchymal transition (EMT). Rutin and related quercetin derivatives suppress EMT by interfering with the TGF-β/Smad and Wnt/β-catenin pathways, down-regulating transcription factors such as Snail, Slug, and Twist, and promoting the re-expression of epithelial markers, thereby reducing migration, invasion, and metastatic potential [38,39]. Kaempferol-3-O-glucoside-3″-rhamnoside and isorhamnetin-3-rutinoside also showed high relative abundance. Kaempferol has been demonstrated to counteract TGF-β1-induced EMT in epithelial and lung cancer cell models by restoring E-cadherin and decreasing N-cadherin and Vimentin expression, thereby attenuating cell migration and invasion [40,41]. Isorhamnetin, a methylated quercetin derivative, inhibits EMT through Nrf2-dependent regulation of AKT/GSK-3β signaling, protecting against both fibrotic and metastatic processes [42]. Procyanidins, namely procyanidin C1 and B1, were also detected. These condensed tannins are well documented to interfere with EMT via EGFR/ERK and TGF-β/Smad signaling. Specifically, procyanidin C1 directly suppresses TGF-β-induced EMT by increasing E-cadherin and decreasing N-cadherin, thereby preventing the acquisition of mesenchymal traits [43]. Coumaric acid derivatives, including coumaroyl hexoside and trans-4-coumaric acid, contributed substantially to the EVs phenolic profile. p-Coumaric acid has been linked to reduced EMT in models of chronic kidney injury [44]. Minor compounds such as methyl gallate and galloyl conjugates are also noteworthy. Methyl gallate inhibits EMT by suppressing Wnt/β-catenin signaling, reducing matrix metalloproteinases and impairing the migratory and invasive capacity of cancer cells [45]. Other metabolites, although less abundant (e.g., citreorosein, biochanin-7-O-glucoside, salicylic acid, p-hydroxybenzaldehyde, corosolic acid), may provide supportive antioxidant and anti-fibrotic activity. For instance, biochanin A, the aglycone of biochanin glucosides, has been shown to attenuate EMT, while anthraquinones such as citreorosein are associated with anti-invasive and anti-fibrotic effects, though with more limited evidence directly linking them to EMT regulation [46,47].

The lipidomic analysis of caper EVs delineates a membrane with a clear phospholipid imprint. Within the glycerophospholipid pool, phosphatidic acid (PA) is markedly enriched and is accompanied by substantial amounts of phosphatidylcholine (PC) and phosphatidylethanolamine (PE) (Figure S3). The most represented molecular species fall within the 16:0/18:X series, with polyunsaturated acyl chains (18:2 and 18:3) recurring across all three classes (e.g., PA 16:0/18:2 and 16:0/18:3; PC 16:0/18:2–18:3; PE 16:0/18:2–18:3) (Table 2). Among neutral glycerolipids, diacylglycerols (DG) and triacylglycerols (TG) are present at low levels, whereas the sphingolipid fraction is modest and dominated by long-chain ceramides. PG 16:0/16:0 occurs at reduced abundance.

Comparison with the broader literature situates this profile within the compositional spectrum reported for plant-derived EVs. Multiple studies and reviews identify PA, PE, and PC as a recurrent triad that constitutes the core of plant EV membranes, with smaller contributions from other classes and species and tissue-dependent variability. Preparations from citrus frequently exhibit elevated PE/PC and comparatively lower PA, whereas vesicles from grape and ginger often display PA-rich signatures [48,49,50,51]. This continuum accommodates the caper EV pattern (high PA with abundant PC and PE) and reinforces the interpretation of a phospholipid-centric architecture. The combination of abundant PA/PE together with high poly-unsaturation supports a bilayer that is quite fluid and curvature-prone, consistent with the mechanical requirements of EV budding and fusion.

Functionally, the predominance of PA and PE provides a mechanistic key to the behavior of these EVs [48]. PA is a cone-shaped lipid with a small, variably charged headgroup that promotes negative curvature [52,53] and acts as a second messenger in plants: it accumulates in response to biotic and abiotic cues, recruits effector proteins, and links membrane remodeling with signal transduction (via convergent PLC/DGK and PLD pathways) [54,55]. PE, also cone-shaped, stabilizes hemifusion intermediates and facilitates the formation of membrane necks during budding and fission/fusion events [56,57]. The high 18:2/18:3 content decreases lateral order and increases bilayer elasticity, lowering energetic barriers to deformation and vesicular traffic. The measurable DG fraction fits within the dynamic DG-PA cycle, indicative of an actively remodeling lipid network during biogenesis; TG likely reflects intraluminal cargo or transient storage. Downstream, converging evidence links the phospholipid signature of plant EVs to tropism and cellular uptake: enrichment in PE and PC has been associated with enhanced entry into epithelial cells [58], while PA and PC differentially modulate interactions with the intestinal environment and microbiota [59,60], thereby influencing distribution along the intestine–liver axis and bacterial engagement [58]. In this framework, the caper EV combination (PA/PE/PC plus PUFA) is compatible with dynamically remodeled membranes and inter-kingdom interactions mediated by biophysical properties and lipid-encoded signals.

### 3.2. C. spinosa EVs Biological Effect on Huh7-EMT Model

#### 3.2.1. *C. spinosa* EV-Enriched Fraction Cellular Uptake

**Figure 2:**
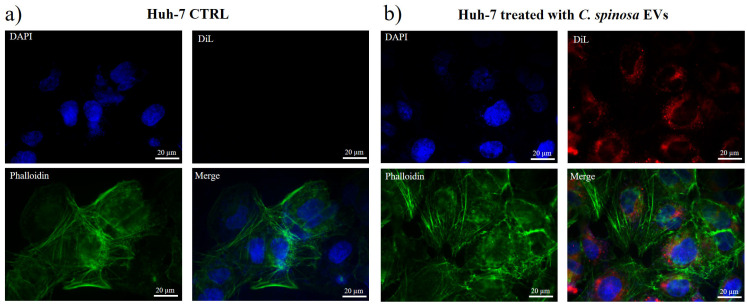
Immunofluorescence analysis of Huh-7 cells treated with *C. spinosa* EV-enriched fraction (**b**) and Huh-7 control (**a**). Nuclei were stained with DAPI (blue), EVs were labeled with the lipophilic dye DiL (red), and the cytoskeleton was visualized with phalloidin (green). EVs DiL-labeled (**b**). Image magnification 60×.

Immunofluorescence analysis was performed to assess the intracellular uptake of the vesicles following the characterization of their molecular markers (Figure 2).

In the fluorescence images, nuclei are stained in blue, DiL-labeled vesicles are shown in red, and the cytoskeleton is visualized in green using phalloidin. The vesicles exhibit a distinct perinuclear accumulation, providing evidence of their internalization and intracellular trafficking. Fluorescence microscopy imaging (Figure 2b) of *C. spinosa* EVs successfully labeled with the lipophilic dye DiL confirms the efficiency of the staining procedure for plant-derived EVs also. Moreover, the absence of any red fluorescence signal in the control (Figure 2a) further confirms the specificity and reliability of the labeling protocol. This observation demonstrates that the DiL dye does not produce nonspecific staining in the absence of EVs, validating that the red signal observed in treated cells indeed corresponds to internalized *C. spinosa*-derived vesicles.

#### 3.2.2. Effect of *C. spinosa* EV-Enriched Fraction on Characteristic Marker of Hepatic EMT Model

Following the characterization of extracellular vesicles isolated from *C. spinosa*, cellular treatments were set up either in combination with the TGF-β growth factor or individually, in order to investigate their effects on the epithelial-to-mesenchymal transition (EMT) process.

**Figure 3:**
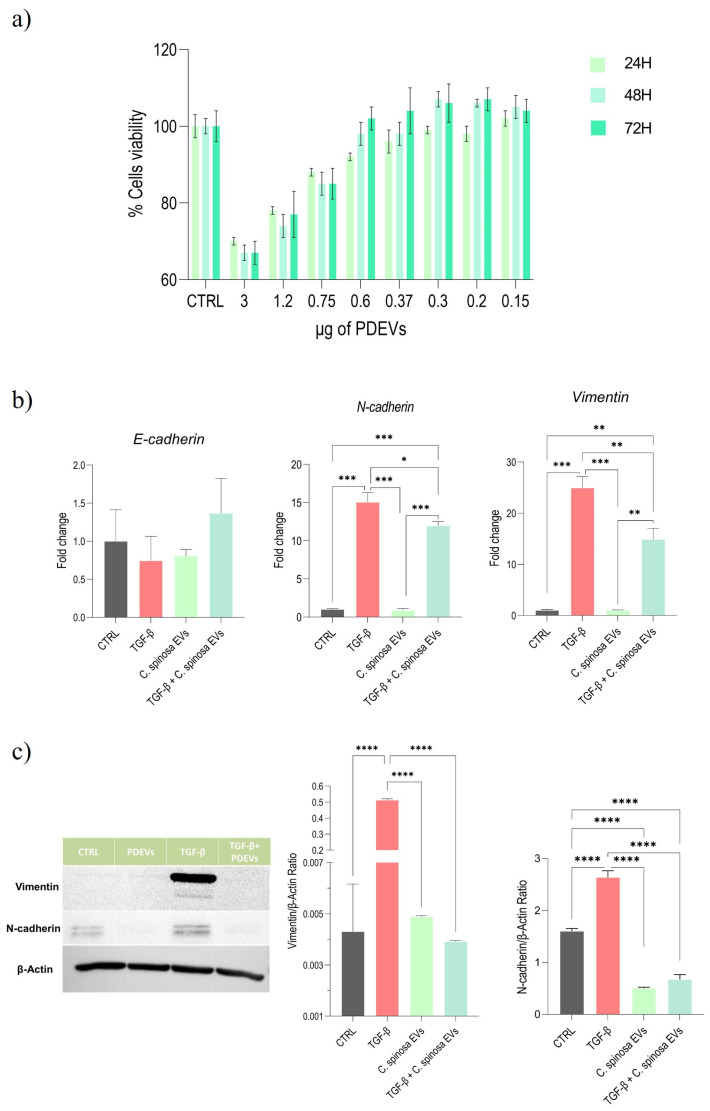
Analysis of *C. spinosa* EVs treatment on the EMT model. (**a**) Cell viability assessed by MTT assay following exposure to different dilutions of PDEVs (1:10–1:200) at 24, 48, and 72 h. Cell viability values were normalized to untreated control cells, set as 100%. Data are expressed as mean ± SD (n = 3). (**b**) Quantitative RT-PCR analysis of epithelial marker E-cadherin and mesenchymal markers N-cadherin and Vimentin following treatments with TGF-β, PDEVs, or their combination. Data are shown as fold change relative to control, normalized to GAPDH (mean ± SD, n = 3). (**c**) Western blot analysis of Vimentin and N-cadherin expression with β-actin as a loading control. Densitometric quantification (right) shows the Vimentin/β-actin ratio and N-cadherin/β-actin ratio (mean ± SD, n = 3). Full, uncropped blots are available in Supplementary Figure S4. Statistical analysis of gene (**b**) and protein (**c**) expression was performed using one-way ANOVA followed by Tukey’s post hoc test, with comparisons conducted among all experimental groups. Only statistically significant differences are indicated in the graphs. Significance levels: * *p* < 0.05, ** *p* < 0.01, *** *p* < 0.001, **** *p* < 0.0001.

To determine the optimal concentration of EVs to be used, cell viability was assessed after exposure to progressive dilutions of PDEVs for 24, 48, and 72 h (Figure 3a).

The most concentrated dilutions (3.0 and 1.2 µg total protein per well, corresponding to former dilutions 1:10 and 1:25) resulted in a marked reduction in cell viability. From the 1:50 dilution onwards (corresponding to a protein content of 0.6 µg), viability remained stable and comparable to the control, and this trend was also maintained at higher dilutions. Based on these results, the 1:50 dilution, corresponding to a protein content of 0.6 µg and a particle number around 7.8 × 10^7^, was selected as the experimental condition for subsequent assays.

Gene expression analysis confirmed that treatment with TGF-β strongly increased mesenchymal markers, as already observed in the previous study (Figure 3b) [17]. Cells treated exclusively with *C. spinosa* EVs displayed very low levels of both genes, comparable to those of the control condition. Co-treatment with TGF-β and EVs resulted in a marked reduction in N-cadherin and Vimentin compared to TGF-β treatment alone, although their expression remained higher than that induced by EVs alone. No significant differences in E-cadherin expression were observed among the different groups.

Protein-level analysis was consistent with the transcriptional results. Immunoblotting demonstrated that treatment with TGF-β induced an increased expression of mesenchymal markers, including Vimentin and N-cadherin, compared to control cells. Treatment with *C. spinosa* EVs alone did not induce more upregulation of these markers than those observed with TGF-β alone. Similarly, the combined treatment with TGF-β and EVs significantly reduced Vimentin and N-cadherin expression compared to TGF-β alone, although levels did not reach those observed in the control or in the EV-only condition (Figure 3c).

#### 3.2.3. Metabolic Profile Huh7 EMT Model Treated with *C. spinosa* EVs

**Figure 4:**
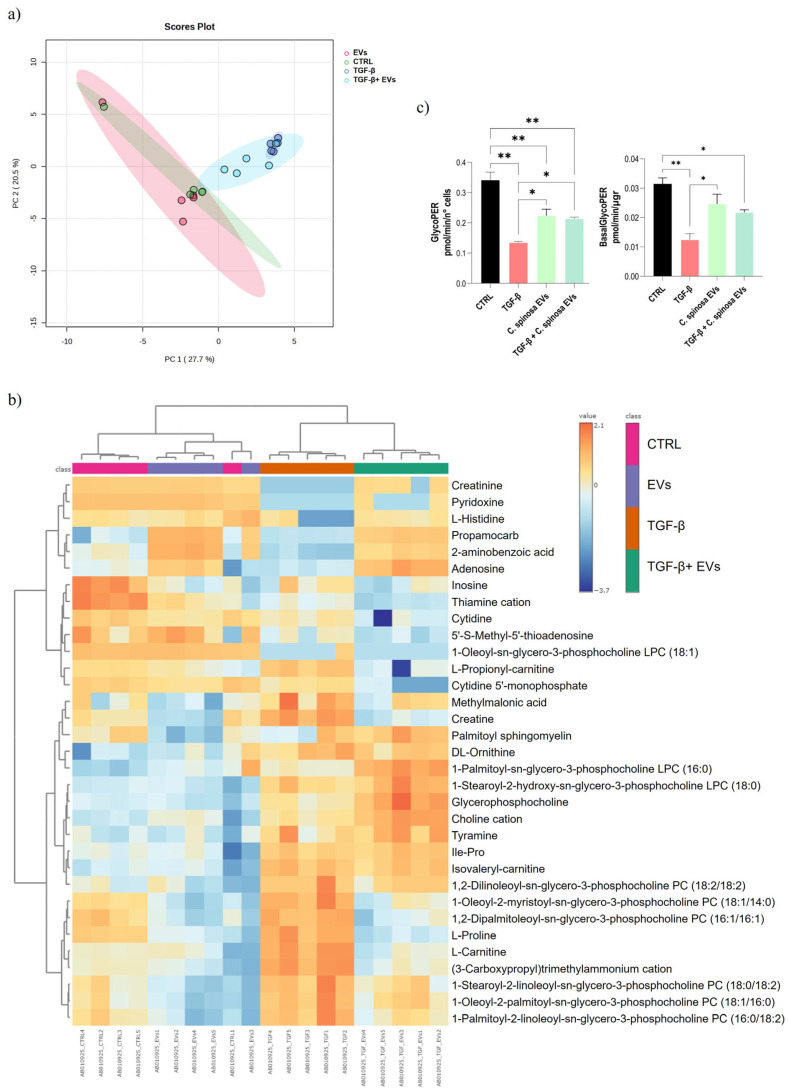
Metabolomic analysis of *C. spinosa* EVs treatment on Huh7-induced EMT. Principal component analysis (PCA) score plot (**a**), hierarchical clustering heatmap (**b**), bar graphs of glycolytic (GlycoPER) and basal mitochondrial (BasalGlycoPER) (**c**). Metabolomic data were normalized by median centering, log-transformed, and Pareto-scaled before multivariate analysis. (**c**) Statistical analysis expression: * *p* < 0.05 and ** *p* < 0.01 (one-way ANOVA followed by Tukey’s post hoc test).

An untargeted metabolomic analysis was also performed to complete the analytical framework of the caper EVs treatment of the in vitro model, which highlighted a significant reorganization of the cellular metabolic profile that involved 71 annotated compounds (Table S1), which have been characterized through MetaboAnalyst tools (Figure 4).

Multivariate analysis by PCA shows that PC1 accounts for 27.7% of the variance and PC2 for 20.5%, explaining together almost 48% of the overall variability (Figure 4a). The CTRL and EVs groups cluster in close proximity, indicating highly similar metabolic profiles and suggesting that extracellular vesicles alone do not substantially alter the basal metabolic state of the hepatic cells. In contrast, the TGF-β group is positioned in a distinct region along PC1, consistent with a marked metabolic reprogramming associated with the induction of EMT and the mesenchymal phenotype. Notably, the TGF-β + EVs group does not overlap with TGF-β alone but occupies an intermediate position, closer to the CTRL/EVs cluster. This distribution does not indicate a sharp separation between groups, but rather points to a modulatory effect of EVs, which appears to attenuate the metabolic shift driven by TGF-β.

The hierarchical clustering analysis presented in the heatmap highlights two main patterns: on one side, CTRL and EVs group closely together, with individual samples even blended, and on the other side, TGF-β and TGF-β + EVs form a separate cluster (Figure 4b). This distribution indicates that EVs, when administered alone, preserve a metabolic state largely indistinguishable from basal conditions, while the presence of TGF-β remains the dominant factor driving global metabolic remodeling. Nevertheless, the observation that TGF-β + EVs in the PCA do not fully overlap with TGF-β alone suggests that EVs introduce a modulatory influence that reshapes, albeit incompletely, the EMT-associated metabolic program. A closer examination of the metabolite distribution suggests that EVs may affect specific subgroups of metabolites with potentially distinct biological implications.

Lipid metabolites showed consistent remodeling upon TGF-β stimulation. Species such as PC (18:0/18:2), PC (18:1/14:0), PC (18:1/16:0), and PC (16:0/18:2) were increased in TGF-β compared with CTRL/EVs, consistent with membrane remodeling processes typically associated with proliferative and fibrotic states. In contrast, caper EVs alone reduced the abundance of these phosphatidylcholines and, in the combined treatment, restored their levels close to those of the control.

This pattern is clearly visible in the heatmap gradient, where the intense red associated with TGF-β is replaced by more neutral tones in the combined condition. Similar trends were observed for PC (16:1/16:1) supporting a modulatory effect of EVs on phospholipid accumulation. In addition, glycerophosphocholine and PC (18:2/18:2) followed a TGF-β-associated increase that was not modulated by EVs, suggesting that not all phosphatidylcholine species are equally responsive to vesicle-mediated normalization. The lysophospholipids (LPC), including LPC (18:0) and LPC (16:0), followed the same TGF-β-associated increase but were not reduced in the combined condition. This behavior is consistent with the role of phospholipid and sphingolipid metabolism in supporting membrane dynamics and motility during EMT, as documented in several studies [61,62].

A distinct behavior was observed for LPC (18:1): this metabolite was abundant in control and EV-treated samples but strongly reduced by TGF-β; in the combined treatment, its levels decreased even further compared with TGF-β alone.

Several metabolites linked to mitochondrial function and cellular energy metabolism displayed coordinated changes across conditions, suggesting an EV-dependent modulation of metabolic stress and remodeling during TGF-β stimulation. L-carnitine, L-propionyl-carnitine and isovaleryl-carnitine followed the same pattern, being higher in TGF-β and normalized in TGF-β + EVs (non-significant decrease for L-isovaleryl-carnitine (Table S1)). Their coordinated behavior across TGF-β and TGF-β + EVs is characteristic of EMT-associated demands on mitochondrial fatty acid beta oxidation (FAO) and membrane remodeling. Multiple studies show that FAO supports TGF-β-induced EMT and invasive behavior; dampening FAO constrains EMT traits [63,64,65]. Consistently, the trajectories of creatinine and methylmalonic acid, both closely associated with cellular energy metabolism and mitochondrial integrity, mirror the patterns described above. Methylmalonic acid was elevated in TGF-β, with a little decrease in TGF-β + EVs, consistent with the mitochondrial remodeling that is influenced by EVs. Creatinine followed the same trajectory, increasing in TGF-β and decreasing in TGF-β + EVs.

Among amino acids and their derivatives, several metabolites displayed condition-specific changes, highlighting potential links between EVs, TGF-β signaling, and metabolic pathways associated with migration and remodeling. DL-ornithine remained elevated under both TGF-β and TGF-β + EVs conditions, indicating a persistent activation of polyamine metabolism, a pathway strongly linked to migration and cytoskeletal reorganization [66]. This sustained elevation suggests that EVs may not counterbalance the polyamine-associated pathways driving invasive traits. The dipeptide Ile-Pro mirrored the behavior of L-proline, showing higher levels under TGF-β treatment. However, whereas proline displayed a decrease attributable to EVs (in combined and singular treatment), the dipeptide did not appear to be influenced by these nanostructures, pointing to a selective modulation of amino acid pools rather than of peptide turnover. The prominence of L-proline fits with the broader literature that places proline metabolism (via PYCR1 and related nodes) at the crossroads of redox management, collagen biosynthesis and migratory programs in tumor and stromal cells engaged in EMT-like remodeling [67,68,69]. L-histidine showed a modest perturbation, reduced in TGF-β and partially restored in TGF-β + EVs, raising the possibility that EVs contribute to buffering histidine availability, potentially influencing nucleotide biosynthesis and histamine-related signaling [70].

For instance, nucleotide-related metabolites and cofactors such as inosine, adenosine and cytidine derivatives appear to be influenced in the EVs condition relative to CTRL, even in the absence of TGF-β. In fact, in the case of adenosine, we observe an increase both in EVs and in the combined treatment compared to the CTRL and to cells treated with TGF-β alone, whereas for inosine a decrease in cluster content is evident, mainly attributable to EVs. Regarding cytidine and its monophosphate, the decrease instead appears to be particularly associated with the combined treatment. These findings are consistent with recent studies indicating that EVs may not only transport nucleotides and purine intermediates as part of their metabolome, but also modulate intracellular metabolic pathways, thereby contributing to the partial attenuation of metabolite pools involved in transcriptional and proliferative programs [71].

Beyond the changes observed in individual metabolites, the bioenergetic profile emerging from the Glycolytic Rate assay (Figure 4c) provides further support. The significant reduction in both GlycoPER and Basal GlycoPER observed in TGF-β-treated cells indeed reflects a decrease in glycolytic metabolism, consistent with the important metabolic reorganization associated with the EMT process and already observed in a previous study [17]. The addition of EVs in combination with TGF-β, on the other hand, led to a partial recovery of both parameters toward CTRL (with significant differences only for GlycoPER), confirming the modulatory effect of the vesicles. This decrease in glycolysis and its partial normalization by EVs are consistent with the distribution of some metabolites related to the energy balance shown in the heatmap, such as creatinine and methylmalonic acid, which reflect alterations in energy metabolism and mitochondrial functionality. These findings strengthen the hypothesis that *C. spinosa* EVs contribute to counteracting the metabolic remodeling induced by TGF-β, partially restoring the cellular bioenergetic balance, but also exert, as observed for other metabolites related to the energy like purine nucleotides (adenosine and inosine), a modulatory effect independent of the EMT process, significantly lowering the measured parameters compared to the control.

#### 3.2.4. Lipidomic Profile Huh7 EMT Model Treated with *C. spinosa* EVs

Lipidomics data were obtained from the same dataset used for the metabolomics analysis of Huh7 cells treated with caper-derived EVs, with the cytokine TGF-β, and with the combination of both treatments. Extraction was performed using a biphasic approach, which enabled the identification and semi-quantification of 87 lipid species, belonging to a total of 13 classes and subclasses. Statistical analysis revealed that eight lipid classes exhibited significant differences attributable to the applied treatments (Table 3 and Table S2).

TGF-β alone induced a marked remodeling of the lipid profile, with significant increases observed in several classes including diacylglycerols (DGs), lysophosphatidylcholines (LPCs), lysophosphatidylethanolamines (LPEs), phosphatidylinositols (PIs), and triglycerides (TGs). The observed lipid classes are associated with distinct cellular processes: DGs act as second messengers in signaling cascades; LPCs and LPEs are involved in membrane remodeling and inflammatory responses; PIs participate in signaling pathways related to cell growth and survival; and TGs reflect alterations in energy storage and metabolic stress. The increase in these lipid classes under TGF-β treatment therefore indicates a lipidomic shift consistent with cellular activation, inflammatory signaling, and pro-fibrotic remodeling. Note that the involvement of these lipid pathways in the EMT is consistent with the findings described in the previous study [17], where the in vitro hepatic model confirmed alterations in some lipid classes. In particular, enhanced membrane turnover, altered signaling through PIs and DGs, and the accumulation of LPCs were observed, supporting their contribution to cytoskeletal reorganization, loss of epithelial polarity, and acquisition of a mesenchymal phenotype. In particular, several lysophospholipid species (LPC 16:0, LPC 18:0, LPC 20:2, LPC 22:6, LPE 18:0 and LPE 18:1) were significantly increased in the TGF-β + EV group compared to the other experimental conditions (Table S2), suggesting enhanced phospholipid turnover under combined treatment.

Moreover, these observations are consistent with the metabolomic dataset, in which LPC and PC species were also found to increase under TGF-β treatment, reinforcing the notion that TGF-β drives a coordinated upregulation of glycerophospholipid metabolism. Treatment with caper-derived EVs alone produced a different profile. In most cases, EVs induced modest or non-significant changes compared to the control, but they did not replicate the pronounced increases elicited by TGF-β. This suggests that EVs per se do not drive lipid remodeling in Huh7 cells.

The combination of EVs with TGF-β produced a distinct effect. In several lipid classes significantly upregulated by TGF-β, such as DG, PI, and TG, co-treatment with EVs attenuated or partially reversed the changes. Notably, Cer levels, which were strongly elevated by TGF-β, were further increased in the combined group, indicating a potential synergistic interaction in this pathway. This suggests a selective modulatory role of EVs, where they may counteract certain pro-lipogenic or pro-inflammatory effects of TGF-β while not affecting (or even enhancing) others.

Multivariate lipidomic analysis revealed a clear discrimination among experimental groups (Figure S5). PLS-DA highlighted the strong remodeling effect induced by TGF-β, while caper-derived EVs alone caused only minor deviations from the control lipid profile. Notably, the combined TGF-β + EVs treatment resulted in an intermediate but distinct lipidomic signature, indicating that EVs modulate, rather than simply reverse, the TGF-β-induced changes. Heatmap analysis (Figure S5b) confirmed these trends, showing a pronounced increase in DG, TG, lysophospholipids and PI species upon TGF-β treatment, which was partially attenuated in the presence of EVs. Overall, these data suggest that caper-derived EVs reshape the TGF-β-driven lipidomic response, steering it toward a partial normalization. Moreover, an integrative correlation analysis between metabolomic and lipidomic profiles highlighted coordinated alterations that, upon pathway enrichment analysis, identified significant modulation of amino acid, nucleotide, and membrane lipid metabolic pathways, further supporting EV-mediated EMT-associated metabolic remodeling (Table S3).

### 3.3. Functional Assessment of Cell Migration

**Figure 5:**
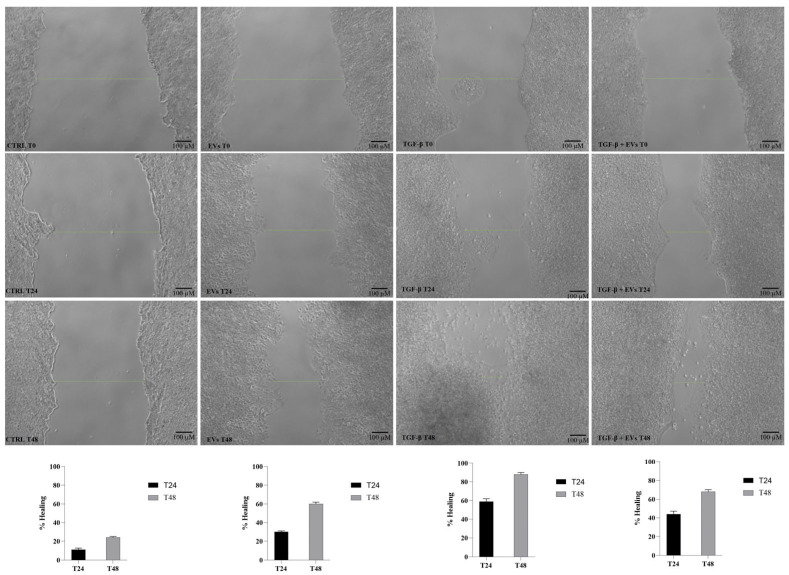
Wound healing assay evaluating the effect of *C. spinosa* EVs on TGF-β-induced cell migration in Huh7 cells. Representative phase-contrast images of scratch closure at 0, 24, and 48 h with scale bar: 100 μm. Quantification of wound closure is shown below as percentage of gap reduction relative to time 0. Data are expressed as mean ± SD (n = 3).

To evaluate whether the modulation of EMT markers was associated with functional changes in cell motility, a wound healing assay was performed (Figure 5).

As expected, TGF-β treatment enhanced wound closure compared to control cells at both 24 and 48 h, with % of remargination of 59 and 88% respectively, confirming the acquisition of increased migratory capacity upon EMT induction. In contrast, treatment with *C. spinosa* EVs alone did not significantly modify wound closure compared to untreated controls, indicating that EVs do not stimulate basal migration. In the co-treatment condition, the combination of TGF-β and EVs resulted in a slight but reproducible reduction in wound closure compared to TGF-β alone at both time points. However, migration remained higher than in control cells, and the attenuation effect was moderate rather than complete.

## 4. Discussion

The results provide new insights into how plant-derived extracellular vesicles influence the epithelial–mesenchymal transition, suggesting that their role is predominantly modulatory rather than strongly inhibitory. Their effect is not to reverse the process and restore a fully epithelial phenotype, but rather to reduce the intensity of the mesenchymal program and to rebalance some of the cellular functions that sustain it. This interpretation is in line with the current understanding of EMT as a spectrum rather than a binary switch.

PDEVs may influence recipient cells through multiple interconnected mechanisms. One key aspect lies in their distinctive lipid composition, enriched in phosphatidic acid, phosphatidylethanolamine, and highly unsaturated phosphatidylcholine species. Such a lipid profile can modulate membrane curvature, fluidity, and fusogenic potential, thereby facilitating interactions with target cell membranes. The incorporation of these lipids into recipient membranes may reorganize lipid microdomains and alter the spatial distribution of signaling complexes, ultimately reshaping how signaling pathways are initiated and sustained. Rather than completely suppressing mesenchymal signaling, these structural perturbations could reduce the efficiency of signal propagation and receptor clustering, leading to a controlled attenuation of EMT-associated signaling activity [72,73,74].

The lipidomic and metabolomic data further support this interpretation. Under TGF-β stimulation, hepatocytes display clear signs of metabolic and lipid remodeling, including increases in diacylglycerols, triglycerides, phosphatidylcholines, and lysophospholipids. These changes are consistent with enhanced signaling activity, membrane turnover, and energetic stress typical of EMT [17]. When *Capparis spinosa* EVs are added, some of these alterations are partially reversed. In particular, the normalization of diacylglycerol and phosphoinositide levels suggests an interference with lipid signaling pathways crucial for maintaining the mesenchymal phenotype [75]. The concurrent restoration of triglyceride content, although not directly related to signaling, likely reflects a re-equilibration of lipid storage and turnover. Triglycerides act as reservoirs for fatty acids and diacylglycerol precursors, and their normalization may indicate a reduced need for lipid remodeling and membrane biosynthesis typically associated with EMT [76,77]. Together, these findings point to a coordinated correction of both structural and signaling lipid pools, ultimately mitigating the metabolic and energetic imbalance driven by TGF-β stimulation [15].

Similarly, the modulation of carnitines, creatinine, and methylmalonic acid by vesicle treatment suggests a relief of mitochondrial metabolic stress and a reduction in fatty acid oxidation pressure, thereby easing the energetic burden imposed by EMT. Under TGF-β stimulation, cells exhibit an increased reliance on lipid catabolism, likely driven by activation of the mitochondrial FAO pathway. Carnitine plays a central role in this process, facilitating the import of long-chain fatty acids into mitochondria via CPT1 and CPT2. Enhanced FAO has been reported to sustain EMT, cellular survival, and invasive behavior across multiple tumor models [78,79,80]. The observed elevation in carnitine derivatives under TGF-β therefore reflects a metabolic rewiring toward intensified lipid utilization to support energy production and biosynthetic demands. However, the contribution of FAO to EMT is context-dependent. In some systems, EMT progression correlates instead with decreased reliance on FAO and a shift toward glycolytic or glutaminolytic metabolism [81]. Moreover, the accumulation of acylcarnitines has been interpreted not only as evidence of increased FAO activity but also as a sign of incomplete β-oxidation or mitochondrial overload [82,83]. These divergent findings indicate that elevated acylcarnitines can reflect either enhanced fatty acid flux or an imbalance between substrate supply and oxidative capacity. In this light, the partial normalization of carnitine and acylcarnitine levels observed in the TGF-β + EVs condition gains particular relevance. It implies that vesicles may buffer the metabolic stress imposed by TGF-β by tempering excessive FAO or improving mitochondrial efficiency, thus constraining the full metabolic reprogramming typically associated with EMT. This interpretation is consistent with the broader trend in the dataset, where EVs appear to attenuate EMT-linked metabolic remodeling.

Not all pathways are equally responsive to modulation by EVs: certain lipid classes appear resilient to reversal to the control level. Lysophospholipids, despite EV treatment, remain elevated, and ceramides even increase relative to TGF-β alone. Given the absence of toxicity at the dosages used, these persistent changes likely represent regulated lipid remodeling rather than cellular stress. Ceramides are well-known to reorganize membrane microdomains and modulate signaling complexes, and their increased accumulation in this context may further restrict migratory dynamics or reinforce signaling checkpoints that inhibit a full EMT [84,85]. Lysophospholipids, on the other hand, often act as bioactive signaling lipids that remain upregulated in cancer and inflammatory contexts, promoting controlled remodeling rather than complete normalization [86,87]. Although our data support EV-associated lipid remodeling and metabolic modulation, direct mechanistic validation was not performed and will require dedicated functional studies.

The wound healing assay provides functional support to the molecular findings. Consistent with the upregulation of mesenchymal markers, TGF-β stimulation increased migratory capacity in Huh7 cells. *C. spinosa* EVs alone did not alter basal migration, suggesting that their effect is not pro- or anti-migratory under physiological conditions, but co-administration of EVs with TGF-β produced a moderate reduction in wound closure compared to TGF-β alone. Although the attenuation was not sufficient to restore migration to control levels, this finding aligns with the observed partial reduction in Vimentin and N-cadherin expression.

**Figure 6:**
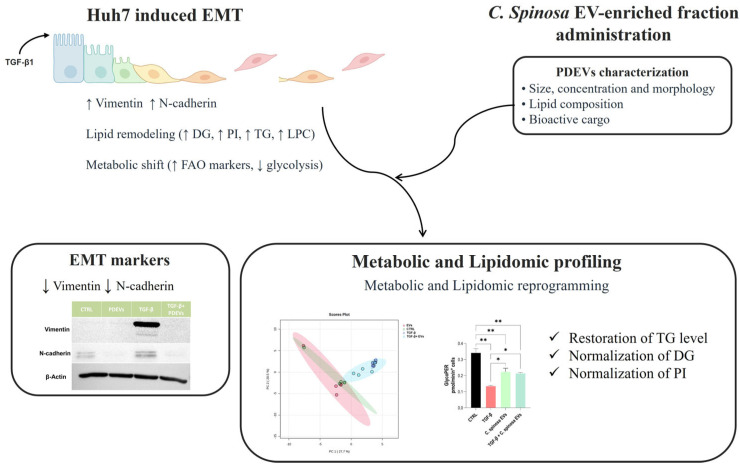
A schematic overview of the study findings. TGF-β1 stimulation induces EMT in Huh7 cells, associated with increased Vimentin and N-cadherin expression, lipid remodeling, and metabolic reprogramming. Treatment with *C. spinosa* EV-enriched fractions attenuates TGF-β-induced mesenchymal marker expression and partially modulates the associated metabolic and lipidomic alterations.

This selective modulation suggests that PDEVs do not globally revert the EMT-associated lipidome but reshape it in a way that weakens pro-mesenchymal outputs while preserving certain adaptive or homeostatic features. Such incomplete reprogramming is consistent with the notion that EVs function as fine-tuners of metabolic signaling rather than as agents of full reversal. A schematic model integrating the molecular, metabolic, lipidomic, and functional findings of this study is presented in Figure 6, illustrating how *C. spinosa* EVs attenuate TGF-β-induced EMT by selectively modulating membrane composition and metabolic signaling rather than fully reversing the mesenchymal state.

While the present study focused on the lipidomic and protein characterization of *C. spinosa* EVs, future investigation of their RNA cargo will be essential to further elucidate the molecular mechanisms underlying their biological activity and their potential contribution to EMT modulation.

## 5. Conclusions

Overall, these findings provide an integrated view of the interplay between EMT and plant-derived extracellular vesicle signaling. They show that the acquisition of mesenchymal traits in hepatocellular carcinoma is accompanied by coordinated structural and metabolic adaptations, and that plant-derived extracellular vesicles can modulate these changes through their distinctive lipid composition and bioactive cargo. Rather than exerting a suppressive effect, PDEVs appear to fine-tune cellular plasticity, promoting the partial attenuation of metabolic balance and membrane homeostasis in a physiologically compatible manner. Beyond their biological relevance, these results support a broader framework for sustainable biomedical innovation. The ability of vesicles derived from edible plants to influence cancer-related pathways highlights a promising convergence between plant biotechnology and nanomedicine, opening new avenues for the development of biocompatible, scalable, and environmentally responsible therapeutic strategies. Further studies will be required to elucidate the molecular determinants of PDEV–cell interactions, define the contribution of specific lipid and phenolic components, and validate these effects in more complex cellular and in vivo models of liver cancer progression.

## Figures and Tables

**Table 1:** Polyphenol species identified in *C. spinosa* NVs through LC/MS.

Metabolites	Means		SD	%
2′,6′-dihydroxy-3,5,7-trihydroxyflavanonol	0.001382	±	0.001933	0.10
benzoyloxyhydroxypropyl-glucuronic acid	0.012964	±	0.007244	0.96
galloylcinnamoylglucose	0.064564	±	0.120196	4.66
galloyl–p-coumaroyl–diglucoside	0.000372	±	0.000693	0.03
1-O-b-D-glucopyranosyl sinapate	0.001696	±	0.001932	0.12
2,5-dihydroxy benzoic acid	0.002113	±	0.002113	0.15
2-Hydroxyphenylacetic acid	0.03235	±	0.024014	2.34
Biochanin-7-O-glucoside	0.001907	±	0.004671	0.14
Citreorosein	0.1003	±	0.134317	7.25
Corosolic acid	0.000123	±	0.0003	0.01
Coumaroyl Hexoside (isomer of 690, 691)	0.113878	±	0.105417	8.24
Isoquercitrin	0.005522	±	0.0069	0.40
isorhamnetin-3-rutinoside	0.097141	±	0.043298	7.02
Kaempferol-3-Glucoside-6″-p-coumaroyl	0.075927	±	0.050394	5.20
Kaempferol-3-O-glucoside-3″-rhamnoside	0.118852	±	0.06192	8.59
Methyl gallate	0.007337	±	0.010345	0.53
p-Hydroxybenzaldehyde	0.020705	±	0.010832	1.50
Procyanidin B1	0.09111	±	0.067935	6.59
Procyanidin C1	0.106271	±	0.104516	7.69
Quercetin-3-O-rutinoside (Rutin)	0.56893	±	0.270082	41.14
Quercitrin	0.008347	±	0.006058	0.60
Salicylic acid	0.012169	±	0.015707	0.88
trans-4-Coumaric acid	0.013711	±	0.014785	0.99
Xanthorhamnin	0.001093	±	0.002676	0.08

**Table 2:** Lipid species identified and quantified in *C. spinosa* EVs through LC/MS.

Lipid Species	Content (nmol/mL)		
PA 16:0_18:1	0.413272	±	0.009639
PA 16:0_18:2	0.513061	±	0.020314
PA 16:0_18:3	0.363506	±	0.014395
PA 18:1_18:1	0.220977	±	0.014578
PA 18:1_18:2	0.08627	±	0.003486
PA 18:2_18:2	0.113408	±	0.005295
PC 16:0_18:1	0.441493	±	0.025465
PC 16:0_18:2	0.400317	±	0.036211
PC 16:0_18:3	0.224835	±	0.008685
PC 18:1_18:1	0.140583	±	0.017354
PE 16:0_18:1	0.196575	±	0.00954
PE 16:0_18:2	0.327014	±	0.014121
PE 16:0_18:3	0.163053	±	0.009659
PE 18:1_18:1	0.075441	±	0.003746
PE 18:1_18:2	0.037174	±	0.003095
DG 16:0_18:2	0.144824	±	0.017573
DG 16:0_18:3	0.116064	±	0.012064
DG 18:2_18:3	0.051893	±	0.040862
TG 16:0_18:1_18:1	0.087131	±	0.005061
TG 18:1_18:1_18:1	0.053777	±	0.002109
Cer 18:1;2O/16:0	0.012913	±	0.000412
Cer 18:1;2O/24:0	0.088473	±	0.005432
SM 8:0;2O/36:8	0.005273	±	0.001564
PG 16:0_16:0	0.079136	±	0.003925

**Table 3:** Lipid classes semi-quantified in CTRL, caper EVs, TGF-β and TGF-β + EVs (mean ± SD, (nmol/mL), n = 5). Statistical analysis was performed using one-way ANOVA followed by Tukey’s post hoc test for multiple pairwise comparisons. The *p*-values reported in the table refer to the ANOVA test. Different lowercase letters (a, b and ab) indicate statistically significant differences between groups (CTRL, EVs, TGF-β and TGF-β + EVs) in the same lipid class. Asterisks indicate the significance level of the overall ANOVA: * *p* < 0.05, ** *p* < 0.01, *** *p* < 0.001.

Class	CTRL		EVs		TGF-β		TGF-β + EVs		S
CL	0.05538 ± 0.02258		0.04680 ± 0.02509		0.02650 ± 0.00271		0.13897 ± 0.05623		
Cer	5.48694 ± 0.77608	b	13.04568 ± 0.8286	b	9.56156 ± 1.47410	b	27.78439 ± 4.43236	a	***
DG	0.14949 ± 0.02324	b	0.21834 ± 0.05418	b	0.50949 ± 0.15996	a	0.16915 ± 0.03756	b	*
LPC	0.72485 ± 0.07036	b	0.83697 ± 0.05414	b	1.03607 ± 0.14274	a	1.49773 ± 0.26688	a	*
LPE	0.39595 ± 0.04807	b	0.40130 ± 0.02427	b	0.41732 ± 0.05429	b	0.99335 ± 0.16605	a	***
PC	55.35140 ± 5.15137		50.37190± 3.28445		72.83965 ± 9.17359		65.12321 ± 14.04254		
PE	16.13464 ± 1.66038	b	17.57489 ± 1.74250	ab	24.25333 ± 2.50377	ab	29.34488 ± 5.24346	a	*
PE-O	0.07897 ± 0.00816	b	0.12018 ± 0.00932	ab	0.11534 ± 0.01133	ab	0.20512 ± 0.04324	a	**
PG	4.07300 ± 0.37995		4.41884 ± 0.35073		5.34345 ± 0.55941		5.65731 ± 1.06083		
PI	9.09941 ± 1.02024	b	8.12896 ± 0.32644	b	16.53078 ± 1.97790	a	12.66614 ± 2.05428	ab	**
PS	0.86256 ± 0.10817		0.82409 ± 0.14015		1.39405 ± 0.12286		1.17542 ± 0.26835		
SM	8.54028 ± 0.83286		7.93844 ± 0.54170		11.66477 ± 1.36306		11.30323 ± 2.26566		
TG	35.45716 ± 3.17500	b	39.69033 ± 7.71265	b	63.57442 ± 9.41239	a	19.40824 ± 4.77285	b	**
Total	136.410 ± 13.0473		143.6167 ± 8.4216		207.2668 ± 26.3601		175.4671 ± 34.3018		

## Data Availability

The original contributions presented in this study are included in the article/Supplementary Materials. Further inquiries can be directed to the corresponding author.

## Supplementary Materials

The following supporting information can be downloaded at: https://www.mdpi.com/article/10.3390/nano16070394/s1, Figure S1. Full, uncropped Western blot images corresponding to Figure 1c,d. Figure S2. Nanoparticle tracking analysis (NTA) size distribution profiles of EV preparations under native conditions (a) and following membrane disruption with Triton X-100 (b). Figure S3. Pie charts show the percentage of lipids Class and Subclass containing in *C. spinosa* EVs. Table S1. The table reports the mean ± SD (N = 5) of the normalized peak areas (median centering, log transformation, and Pareto scaling) of metabolic LC/MS dataset. Table S2. The table reports the mean ± SD (N = 5) of each lipid species quantified in the lipidomic dataset across three experimental conditions: CTRL, caper derived-EVs, TGF-β1 10 ng/mL, and TGF-β1 + EVs. Figure S4. Full, uncropped Western blot images corresponding to Figure 3c. Figure S5. Lipidomic Analysis of caper EVs treatment of Huh7-induced EMT. Table S3. Enriched metabolic pathways identified from integrative metabolomic–lipidomic correlation analysis. Pathways are ranked according to statistical significance and topology-based impact score; raw p-values, Holm correction, and FDR-adjusted values are reported.

## Abbreviations

The following abbreviations are used in this manuscript:

2-DG	2-deoxy-D-glucose
AF	Apoplastic fluid
Cer	Ceramides
CL	Cardiolipins
DAPI	4′,6-diamidino-2-phenylindole
DG	Diacylglycerols
DiL	1,1′-dioctadecyl-3,3,3′,3′-tetramethylindocarbocyanine perchlorate
EGFR	Epidermal growth factor receptor
EMT	Epithelial-to-mesenchymal transition
ERK	Extracellular signal-regulated kinase
EVs	Extracellular vesicles
FAO	Fatty acid oxidation
GAPDH	Glyceraldehyde-3-phosphate dehydrogenase
GlycoPER	Glycolytic proton efflux rate
GSK-3β	Glycogen synthase kinase 3 beta
HCC	Hepatocellular carcinoma
HGF	Hepatocyte growth factor
HRP	Horseradish peroxidase
IL-6	Interleukin 6
LPC	Lysophosphatidylcholines
LPE	Lysophosphatidylethanolamines
Nrf2	Nuclear factor erythroid 2–related factor 2
NTA	Nanoparticle tracking analysis
PA	Phosphatidic acid
PC	Phosphatidylcholines
PCA	Principal component analysis
PDEVs	Plant-derived extracellular vesicles
PE	Phosphatidylethanolamines
PE-O	Ether phosphatidylethanolamines
PER	Proton efflux rate
PG	Phosphatidylglycerols
PI	Phosphatidylinositols
PLD	Phospholipase D
PLC	Phospholipase C
PLS-DA	Partial least squares–discriminant analysis
PS	Phosphatidylserines
PUFA	Polyunsaturated fatty acids
PVDF	Polyvinylidene difluoride
Rot/AA	Rotenone/antimycin A
SEM	Scanning electron microscopy
SLC27	Solute carrier family 27
Smad	SMAD family proteins
SM	Sphingomyelin(s)
TGF-β	Transforming growth factor beta
TG	Triglycerides
TPC	Total phenolic content
Wnt	Wingless-related integration site
