# Supplementary material for: Extracellular Vesicles from Capparis spinosa Modulate Epithelial-to-Mesenchymal Transition in Huh7 Hepatocellular Carcinoma Cells
Source: Nanomaterials (Basel). 2026 Mar 25;16(7):394. doi: 10.3390/nano16070394 (PMC13074539; doi:10.3390/nano16070394)
Supplement: Supplementary file 1 [file nanomaterials-16-00394-s001.zip › nanomaterials-4155424-supplementary.pdf]

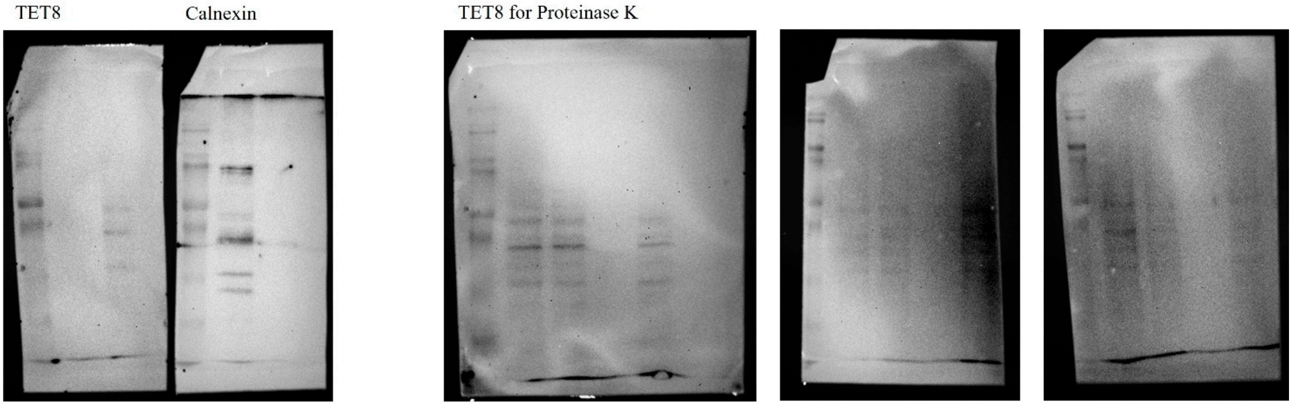


**Figure S1.** Full, uncropped Western blot images corresponding to Figure 1c and 1d.

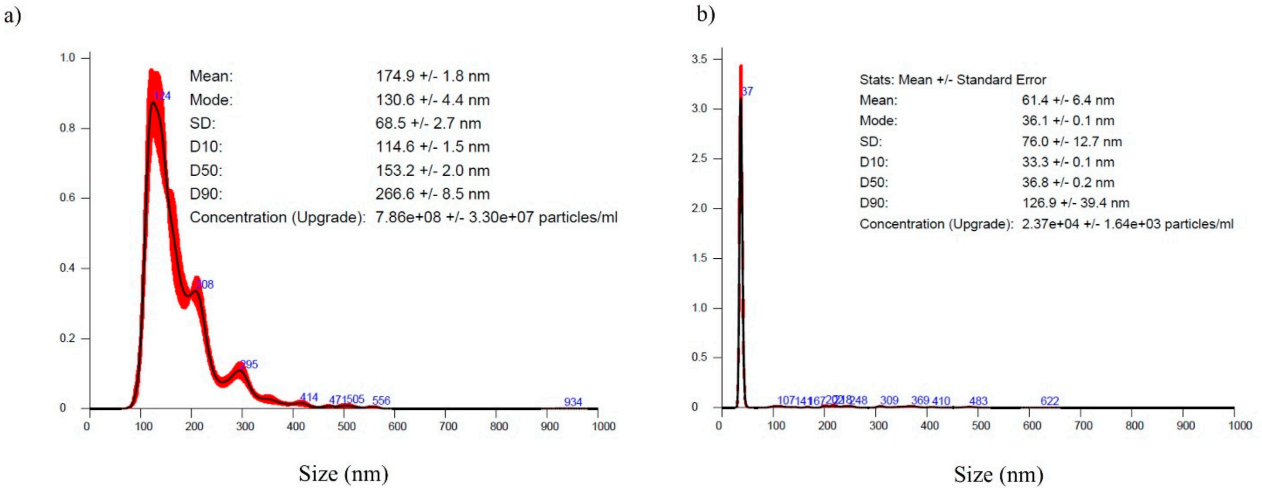


**Figure S2.** Nanoparticle tracking analysis (NTA) size distribution profiles of EV preparations under native conditions (a) and following membrane disruption with Triton X-100 (b).

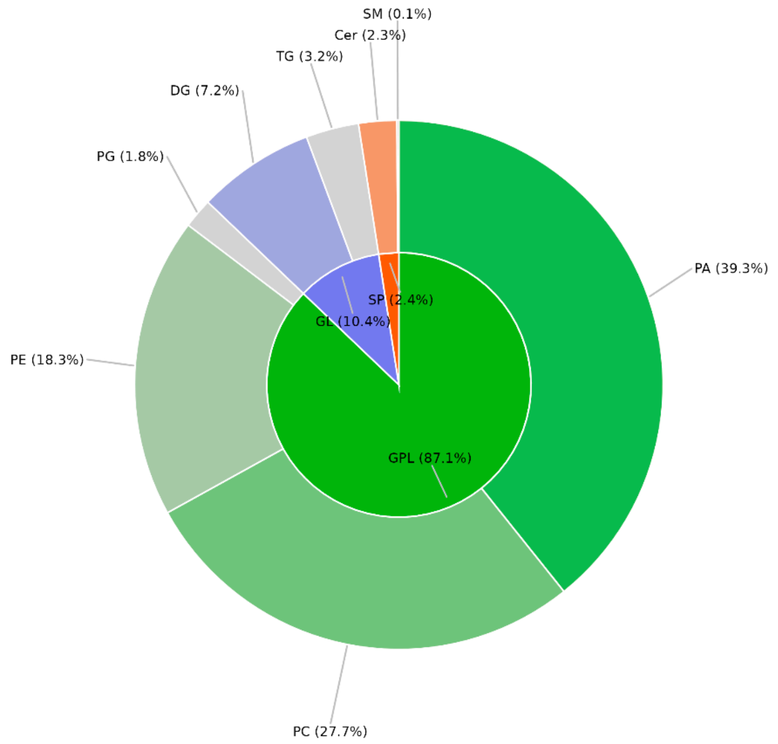


**Figure S3.** Pie charts show the percentage of lipids Class and Subclass containing in *C. spinosa* EVs.

**Table S1.** The table reports the mean ± SD (N = 5) of the normalized peak areas (median centering, log transformation, and Pareto scaling) of metabolic LC/MS dataset. For each variable the compared condition pairs, unadjusted p-values, and FDR-adjusted p-values are reported. Statistically significant differences (FDR < 0.05) indicate alterations associated with TGF-β and EVs treatment. Results of multiple comparisons between experimental conditions (denoted as A, B, C and D respectively CTRL, EVs, TGF-β 10 ng/mL, and TGF-β+EVs) using Tukey's HSD test.

| | **CTRL (A)** | **EVs (B)** | **TGF-β (C)** | **TGF-β+EVs (D)** | | | |
|---|---|---|---|---|---|---|---|
| Label | Mean ± SD | Mean ± SD | Mean ± SD | Mean ± SD | p.value | FDR | Tukey's HSD |
| (3-Carboxypropyl) trimethyl ammonium cation | -0.182 ± 0.413 | -0.425 ± 0.368 | 0.745 ± 0.140 | -0.137 ± 0.205 | 8.61E-05 | 0.00033 | C-A; C-B; D-C |
| (R)-Butyrylcarnitine | 0.032 ± 0.526 | 0.145 ± 0.523 | 0.177 ± 0.106 | -0.354 ± 0.248 | | | |
| .alpha.-D-Mannose 1-phosphate | 0.078 ± 1.351 | 0.527 ± 1.529 | -0.078 ± 1.004 | -0.527 ± 0.116 | | | |
| 1,2-Dilinoleoyl-sn-glycero-3-PC | -0.311 ± 0.343 | -0.393 ± 0.390 | 0.447 ± 0.205 | 0.257 ± 0.193 | 0.000633 | 0.00182 | C-A D-A; C-B; D-B |
| 1,2-Dipalmitoleoyl-sn-glycero-3-PC | 0.276 ± 0.251 | -0.557 ± 0.273 | 0.617 ± 0.121 | -0.336 ± 0.334 | 5.87E-06 | 3.68E-05 | B-A; D-A; C-B; D-C |
| 1,5-Pentanediamine | 0.027 ± 0.620 | 0.001 ± 0.183 | 0.219 ± 0.236 | -0.246 ± 0.269 | | | |
| 1-Methylnicotinamide cation | -0.334 ± 0.570 | 0.014 ± 0.542 | -0.134 ± 0.142 | 0.454 ± 0.152 | | | |
| 1-Oleoyl-2-myristoyl-sn-glycero-3-PC | 0.121 ± 0.267 | -0.601 ± 0.280 | 0.641 ± 0.178 | -0.162 ± 0.272 | 8.05E-06 | 4.63E-05 | B-A; C-A; C-B; D-C |
| 1-Oleoyl-2-palmitoyl-sn-glycero-3-PC | -0.077 ± 0.344 | -0.585 ± 0.263 | 0.487 ± 0.191 | 0.175 ± 0.224 | 6.58E-05 | 0.000267 | B-A; C-A; C-B; D-B |
| 1-Oleoyl-sn-glycero-3-PC | -0.307 ± 0.583 | 0.183 ± 0.130 | 0.028 ± 0.818 | 0.096 ± 0.790 | | | |
| 1-Palmitoyl-2-linoleoyl- | 0.045 ± 0.325 | -0.606 ± 0.358 | 0.500 ± 0.207 | 0.061 ± 0.186 | 0.000128 | 0.000441 | B-A; C-B; |

| | | | | | | | |
|---|---|---|---|---|---|---|---|
| sn-glycero-3-PC | | | | | | | D-B |
| 1-Palmitoyl-sn-glycero-3-PC | -0.739 ± 0.340 | -0.106 ± 0.533 | -0.026 ± 0.083 | 0.871 ± 0.145 | 9.86E-06 | 5.23E-05 | B-A; C-A; D-A; D-B; D-C |
| 1-Piperidinecarbaldehyde | -0.667 ± 0.873 | 0.195 ± 0.155 | 0.204 ± 0.241 | 0.268 ± 0.671 | | | |
| 1-Stearoyl-2-arachidonoyl-sn-glycero-3-phosphoserine | 0.287 ± 1.355 | 0.349 ± 1.493 | -0.318 ± 0.004 | -0.318 ± 0.060 | | | |
| 1-Stearoyl-2-hydroxy-sn-glycero-3-PC | -0.530 ± 0.355 | -0.458 ± 0.185 | 0.337 ± 0.181 | 0.651 ± 0.256 | 1.90E-06 | 1.87E-05 | C-A; D-A; C-B; D-B |
| 1-Stearoyl-2-linoleoyl-sn-glycero-3-PC | -0.052 ± 0.358 | -0.602 ± 0.286 | 0.530 ± 0.220 | 0.125 ± 0.239 | 9.71E-05 | 0.000353 | B-A; C-A; C-B; D-B |
| 2,3-Dihydroxypropyl dihydrogen phosphate | -0.301 ± 0.624 | -0.217 ± 0.945 | 0.209 ± 0.206 | 0.309 ± 0.242 | | | |
| Tyramine | -0.388 ± 0.313 | -0.261 ± 0.182 | 0.226 ± 0.292 | 0.424 ± 0.205 | 0.000272 | 0.000895 | C-A; D-A; C-B; D-B |
| 2-Aminoethyl dihydrogen phosphate | 0.069 ± 0.801 | 0.123 ± 1.352 | -0.315 ± 1.293 | 0.123 ± 1.264 | | | |
| Adenosine | -0.400 ± 0.529 | 0.211 ± 0.446 | -0.447 ± 0.113 | 0.636 ± 0.137 | 0.000447 | 0.00134 | D-A; C-B; D-C |
| 4-Imidazoleacrylic acid | -0.338 ± 0.546 | -0.100 ± 0.534 | 0.071 ± 0.236 | 0.367 ± 0.328 | | | |
| 5'-S-Methyl-5'-thioadenosine | 0.116 ± 0.521 | 0.418 ± 0.197 | -0.124 ± 0.212 | -0.410 ± 0.158 | 0.004419 | 0.010514 | D-B |
| Acetyl-L-carnitine | 0.053 ± 0.596 | -0.099 ± 0.559 | 0.271 ± 0.159 | -0.224 ± 0.215 | | | |
| Adenosine 5'-monophosphate | 0.245 ± 1.389 | -0.122 ± 1.431 | -0.721 ± 0.738 | 0.598 ± 0.926 | | | |
| Isovaleryl-carnitine | -0.587 ± 0.466 | -0.466 ± 0.353 | 0.580 ± 0.122 | 0.473 ± 0.163 | 1.36E-05 | 6.68E-05 | C-A; D-A; C-B; D-B |
| Betaine | 0.099 ± 0.777 | -0.308 ± 0.574 | 0.102 ± 0.295 | 0.106 ± 0.192 | | | |
| Choline cation | -0.729 ± 0.445 | -0.253 ± 0.355 | 0.178 ± 0.107 | 0.804 ± 0.186 | 4.89E-06 | 3.38E-05 | C-A; D-A; D-B; D-C |
| Creatine | 0.179 ± 0.124 | -0.483 ± 0.353 | 0.688 ± 0.136 | -0.384 ± 0.204 | 9.37E-07 | 1.29E-05 | B-A; C-A; D-A; C-B; D-C |
| Creatinine | 1.081 ± 0.164 | 1.061 ± 0.134 | -2.325 ± 0.002 | 0.183 ± 1.403 | 2.40E-06 | 2.07E-05 | C-A; C-B; D-C |
| Cytidine | 0.695 ± 0.127 | 0.285 ± 0.075 | 0.141 ± 0.174 | -1.121 ± 1.407 | 0.005 | 0.013641 | D-A; D-B |
| Cytidine 5'-monophosphate | 0.863 ± 0.135 | 0.520 ± 0.189 | 0.421 ± 0.199 | -1.804 ± 1.085 | 4.37E-06 | 3.35E-05 | D-A; D-B; D-C |
| D-Lyxose | 0.060 ± 0.363 | 0.438 ± 0.326 | -0.337 ± 0.425 | -0.160 ± 0.908 | | | |
| D-Ribose 5-phosphate | -0.231 ± 1.287 | 0.422 ± 1.194 | 0.123 ± 1.274 | -0.313 ± 1.104 | | | |
| D-erythro-Sphinganine | -0.259 ± 0.799 | -0.332 ± 0.869 | 0.207 ± 0.115 | 0.384 ± 0.316 | | | |
| DL-Ornithine | -0.646 ± 0.572 | -0.296 ± 0.472 | 0.504 ± 0.300 | 0.438 ± 0.209 | 0.00087 | 0.002309 | C-A; D-A; C-B |
| DL-Phenylalanine | -0.290 ± 0.622 | -0.204 ± 0.208 | 0.433 ± 0.250 | 0.061 ± 0.159 | | | |
| Gentianose | 0.231 ± 1.169 | 0.353 ± 1.441 | -0.292 ± 0.033 | -0.292 ± 0.033 | | | |
| Glycerophosphocholine | -0.472 ± 0.296 | -0.464 ± 0.148 | 0.111 ± 0.117 | 0.825 ± 0.280 | 1.85E-07 | 4.25E-06 | C-A; D-A; C-B; D-B; D-C |
| Hypoxanthine | -0.255 ± 0.566 | -0.317 ± 0.459 | 0.175 ± 0.165 | 0.397 ± 0.206 | | | |
| Indacaterol | 0.027 ± 0.941 | 0.003 ± 1.384 | 0.004 ± 1.350 | -0.034 ± 1.292 | | | |
| Indole-2-carboxylic acid | -0.087 ± 1.128 | 0.075 ± 1.344 | 0.078 ± 0.222 | -0.067 ± 0.238 | | | |
| Inosine | 0.456 ± 0.279 | -0.246 ± 0.266 | 0.041 ± 0.270 | -0.251 ± 0.256 | 0.002045 | 0.005039 | B-A; D-A |
| 1-Oleoyl-sn-glycero-3-phosphocholine (18:1) | 1.397 ± 0.101 | 1.339 ± 0.125 | -1.103 ± 1.186 | -1.633 ± 0.135 | 2.66E-07 | 4.58E-06 | C-A; D-A; C-B; D-B |

| | | | | | | | |
|---|---|---|---|---|---|---|---|
| L-Arginine | 0.161 ± 0.764 | -0.340 ± 0.881 | 0.043 ± 0.546 | 0.136 ± 0.282 | | | |
| L-Carnitine | -0.217 ± 0.442 | -0.264 ± 0.426 | 0.701 ± 0.155 | -0.221 ± 0.235 | 0.000727 | 0.002006 | C-A; C-B; D-C |
| L-Citrulline | -0.248 ± 0.811 | 0.019 ± 1.333 | 0.107 ± 0.203 | 0.122 ± 0.095 | | | |
| L-Glutamic acid | -0.472 ± 1.491 | -0.427 ± 1.574 | 0.750 ± 0.219 | 0.148 ± 1.171 | | | |
| L-Glutamine | 0.049 ± 0.376 | 0.186 ± 0.667 | 0.294 ± 0.328 | -0.529 ± 0.594 | | | |
| L-Histidine | 0.515 ± 0.334 | 0.572 ± 0.432 | -1.396 ± 1.674 | 0.309 ± 0.123 | 0.007794 | 0.017347 | C-A; C-B; D-C |
| L-Leucine | -0.082 ± 0.639 | 0.069 ± 0.132 | 0.268 ± 0.295 | -0.255 ± 0.247 | | | |
| L-Lysine | 0.295 ± 0.667 | -0.599 ± 1.740 | -0.186 ± 1.211 | 0.489 ± 0.110 | | | |
| L-Proline | 0.193 ± 0.403 | -0.389 ± 0.313 | 0.649 ± 0.141 | -0.453 ± 0.170 | 2.58E-05 | 0.000119 | B-A; D-A; C-B; D-C |
| L-Propionyl-carnitine | 0.216 ± 0.282 | -0.061 ± 0.303 | 0.615 ± 0.110 | -0.770 ± 1.041 | 0.00942 | 0.019695 | D-C |
| L-Serine | 0.335 ± 0.434 | 0.246 ± 0.462 | -0.018 ± 1.300 | -0.564 ± 1.619 | | | |
| Methylmalonic acid | -0.042 ± 0.248 | -0.297 ± 0.245 | 0.312 ± 0.238 | 0.026 ± 0.217 | 0.008287 | 0.01787 | C-B |
| N-Acetyl-D-glucosamine | 0.485 ± 0.210 | 0.045 ± 0.758 | 0.246 ± 0.253 | -0.776 ± 1.402 | | | |
| N-Acetyl-L-aspartic acid | 0.128 ± 0.426 | -0.799 ± 1.526 | 0.443 ± 0.437 | 0.229 ± 0.131 | | | |
| N-Acetyl-L-glutamic acid | 0.137 ± 0.559 | -0.391 ± 1.267 | 1.023 ± 0.124 | -0.769 ± 1.507 | | | |
| N-Desmethyltamoxifen | -0.291 ± 0.560 | -0.421 ± 1.172 | 0.196 ± 0.363 | 0.515 ± 1.068 | | | |
| NG,NG-Dimethyl-L-arginine | 0.370 ± 1.649 | 0.363 ± 1.633 | -0.367 ± 0.001 | -0.367 ± 0.135 | | | |
| Nicotinamide riboside cation | -0.140 ± 0.575 | -0.175 ± 0.495 | 0.412 ± 0.206 | -0.097 ± 0.224 | | | |
| Palmitoyl sphingomyelin | 0.165 ± 0.185 | -0.633 ± 0.358 | 0.001 ± 0.438 | 0.467 ± 0.188 | 0.000351 | 0.001101 | B-A; C-B; D-B |
| Pantothenic acid | -0.012 ± 0.596 | 0.288 ± 0.426 | -0.120 ± 0.180 | -0.155 ± 0.184 | | | |
| Ile-Pro | -0.562 ± 0.781 | -0.463 ± 0.506 | 0.480 ± 0.116 | 0.545 ± 0.148 | 0.001947 | 0.004975 | C-A; D-A; C-B; D-B |
| Propamocarb | -1.001 ± 0.762 | 1.162 ± 0.226 | -1.046 ± 0.071 | 0.884 ± 0.086 | 6.12E-08 | 2.11E-06 | B-A; D-A; C-B; D-C |
| Pyridoxine | 1.367 ± 0.144 | 1.361 ± 0.137 | -1.859 ± 0.120 | -0.869 ± 1.359 | 1.32E-06 | 1.52E-05 | C-A; D-A; C-B; D-B |
| Tamoxifen | 0.030 ± 1.493 | 0.085 ± 1.609 | -0.652 ± 1.283 | 0.536 ± 1.298 | | | |
| Thiamine cation | 0.682 ± 0.413 | 0.027 ± 0.373 | -0.139 ± 0.113 | -0.570 ± 0.142 | 4.89E-05 | 0.000211 | B-A; C-A; D-A; D-B |
| 2-aminobenzoic acid | -0.309 ± 0.365 | 0.985 ± 0.187 | -1.217 ± 0.253 | 0.542 ± 0.117 | 9.96E-10 | 6.87E-08 | B-A; C-A; D-A; C-B; D-C |

**Table S2.** The table reports the mean ± SD (N = 5) of each lipid species quantified in the lipidomic dataset across three experimental conditions: CTRL, caper derived-EVs, TGF-β1 10 ng/mL, and TGF-β1+EVs. Lipid abundances were normalized (median centering, log transformation, and Auto- scaling) and compared using one-way ANOVA followed by Tukey's HSD post hoc test. For each species, unadjusted and FDR-adjusted p-values are provided. Results of multiple comparisons between experimental conditions (denoted as A, B, C and D respectively CTRL, EVs, TGF-β1 , and TGF-β1+EVs) using Tukey's HSD test.

| Lipid | CTRL (A) Mean ± SD | EVs (B) Mean ± SD | TGF-β (C) Mean ± SD | TGF-β+B (D) Mean ± SD | p.value | FDR | Tukey's HSD |
|---|---|---|---|---|---|---|---|
| DG 16:0_18:1 | 0.040291 ± 0.005483 | 0.046616 ± 0.023495 | 0.155466 ± 0.108431 | 0.047684 ± 0.017929 | 0.013707 | 0.017283 | A-C, C-D |
| DG 16:0_18:2 | 0.030232 ± 0.012546 | 0.05171 ± 0.031392 | 0.197924 ± 0.115962 | 0.034973 ± 0.017547 | 0.000174 | 0.000329 | A-C, B-C, C-D |
| DG 18:1_18:1 | 0.061089 ± 0.024357 | 0.090728 ± 0.063369 | 0.225624 ± 0.148481 | 0.106162 ± 0.057663 | 0.622121 | 0.629355 | |
| CL 16:0_18:1_ | 0.062091 ± 0.05566 | 0.055706 ± 0.060572 | 0.026439 ± 0.007007 | 0.163324 ± 0.13085 | 0.015206 | 0.018373 | C-D |

| | | | | | | | | | | | | | | | |
|---|---|---|---|---|---|---|---|---|---|---|---|---|---|---|---|
| 16:0_18:1 | | | | | | | | | | | | | | | |
| Cer 18:1;2O/16:0 | 3.82894 | ± | 0.855926 | 11.23999 | ± | 1.263234 | 8.072493 | ± | 2.797417 | 23.83041 | ± | 9.371748 | 1.91E-12 | 2.77E-11 | A-B, A-D, B-C, B-D, C-D |
| Cer 18:1;2O/18:1 | 0.17408 | ± | 0.027895 | 0.5441 | ± | 0.147669 | 0.462327 | ± | 0.14403 | 1.005872 | ± | 0.39562 | 1.29E-10 | 9.75E-10 | A-B, A-C, A-D, B-C, B-D, C-D |
| Cer 18:1;2O/24:1 | 0.84425 | ± | 0.264591 | 1.711097 | ± | 0.579939 | 1.748988 | ± | 0.380409 | 3.844896 | ± | 1.444894 | 3.56E-09 | 1.72E-08 | A-B, A-D, B-C, B-D, C-D |
| LPC 16:0 | 0.537301 | ± | 0.13229 | 0.567743 | ± | 0.047841 | 0.729663 | ± | 0.225898 | 0.946866 | ± | 0.391089 | 5.56E-10 | 3.22E-09 | A-C, A-D, B-C, B-D, C-D |
| LPC 18:0 | 0.148436 | ± | 0.036163 | 0.299407 | ± | 0.033269 | 0.343167 | ± | 0.10274 | 0.591643 | ± | 0.248759 | 1.44E-14 | 6.26E-13 | A-B, A-C, A-D, B-C, B-D, C-D |
| LPC 20:2 | 0.011187 | ± | 0.004803 | 0.007493 | ± | 0.004577 | 0.011687 | ± | 0.002062 | 0.024321 | ± | 0.011616 | 0.000533 | 0.000813 | B-D, C-D |
| LPC 22:6 | 0.009119 | ± | 0.002693 | 0.007414 | ± | 0.001417 | 0.011771 | ± | 0.003979 | 0.017874 | ± | 0.003928 | 0.005837 | 0.007468 | B-D, C-D |
| LPE 18:0 | 0.220721 | ± | 0.067466 | 0.275202 | ± | 0.023695 | 0.283081 | ± | 0.084399 | 0.629452 | ± | 0.249585 | 2.97E-12 | 3.69E-11 | A-B, A-C, A-D, B-C, B-D, C-D |
| LPE 18:1 | 0.170756 | ± | 0.056217 | 0.145139 | ± | 0.01554 | 0.159616 | ± | 0.039568 | 0.421631 | ± | 0.153779 | 3.58E-15 | 3.12E-13 | A-B, A-C, A-D, B-C, B-D, C-D |
| PC 16:0_16:0 | 6.791937 | ± | 1.110739 | 6.110359 | ± | 0.761464 | 7.96405 | ± | 2.576398 | 6.721353 | ± | 3.160203 | 0.0002 | 0.000362 | A-C, A-D, B-C |
| PC 16:0_16:1 | 7.756141 | ± | 1.178349 | 5.287846 | ± | 0.809704 | 13.7194 | ± | 4.352312 | 7.43382 | ± | 3.211456 | 4.89E-08 | 1.93E-07 | A-B, A-D, B-C, B-D, C-D |
| PC 16:0_18:0 | 2.314063 | ± | 0.416454 | 4.419201 | ± | 1.567062 | 3.235271 | ± | 0.968158 | 6.152324 | ± | 2.753241 | 8.04E-09 | 3.68E-08 | A-B, A-D, B-C, C-D |
| PC 16:0_18:1 | 16.94955 | ± | 3.168851 | 16.10632 | ± | 2.121363 | 23.3988 | ± | 7.077971 | 23.49193 | ± | 10.58826 | 0.258122 | 0.26734 | |
| PC 16:0_20:4 | 2.515081 | ± | 0.389163 | 4.383145 | ± | 0.612195 | 5.065071 | ± | 1.514192 | 5.713045 | ± | 2.737711 | 2.84E-08 | 1.18E-07 | A-B, A-C, A-D, B-C, C-D |
| PC 16:0_22:6 | 0.885697 | ± | 0.151865 | 0.749908 | ± | 0.128722 | 1.490759 | ± | 0.454656 | 0.65336 | ± | 0.275462 | 1.94E-08 | 8.44E-08 | A-B, A-D, B-C, B-D, C-D |
| PC 18:0_18:1 | 4.567398 | ± | 0.801412 | 5.951355 | ± | 2.140776 | 6.688675 | ± | 1.969592 | 7.633355 | ± | 3.260034 | 0.000624 | 0.000904 | A-D, B-C, C-D |
| PC 18:0_20:3 | 1.238715 | ± | 0.214497 | 0.780554 | ± | 0.263637 | 2.731024 | ± | 0.824781 | 1.060434 | ± | 0.431199 | 0.000261 | 0.000437 | A-B, B-C, C-D |
| PC 18:1_18:1 | 7.221339 | ± | 1.311827 | 6.052032 | ± | 1.087543 | 9.230946 | ± | 2.637278 | 9.842904 | ± | 4.414341 | 9.86E-06 | 2.45E-05 | A-B, A-C, B-D, C-D |
| PC 18:1_20:4 | 1.259512 | ± | 0.153434 | 1.972016 | ± | 0.235977 | 1.70991 | ± | 0.526416 | 2.91396 | ± | 1.342766 | 1.22E-10 | 9.75E-10 | A-B, A-C, A-D, B-C, B-D, C-D |
| PE 16:0_18:1 | 0.662132 | ± | 0.157146 | 1.091266 | ± | 0.272468 | 0.932884 | ± | 0.261878 | 1.794477 | ± | 0.680306 | 2.79E-11 | 3.04E-10 | A-B, A-C, A-D, B-C, B-D, C-D |
| PE 16:0_20:3 | 0.700695 | ± | 0.206683 | 0.347588 | ± | 0.059251 | 1.500512 | ± | 0.335449 | 0.563091 | ± | 0.210391 | 0.173161 | 0.181506 | |
| PE 16:0_20:4 | 1.435628 | ± | 0.283888 | 1.442219 | ± | 0.170332 | 2.39054 | ± | 0.537308 | 1.889454 | ± | 0.711973 | 0.796473 | 0.796473 | |
| PE 16:0_20:5 | 0.173361 | ± | 0.031801 | 0.143157 | ± | 0.017188 | 0.267201 | ± | 0.066478 | 0.204484 | ± | 0.077042 | 0.058778 | 0.06556 | |
| PE 16:0_22:6 | 0.775222 | ± | 0.17205 | 0.671588 | ± | 0.094462 | 1.600341 | ± | 0.370264 | 0.893524 | ± | 0.333591 | 0.000257 | 0.000437 | A-B, B-C, C-D |

| | | | | | | | |
|---|---|---|---|---|---|---|---|
| PE 16:1_18:1 | 0.221419 ± 0.048983 | 0.212161 ± 0.027565 | 0.277024 ± 0.068398 | 0.405464 ± 0.158039 | 1.75E-06 | 5.07E-06 | A-C, A-D, B-D, C-D |
| PE 18:0_18:1 | 0.50886 ± 0.129924 | 1.08122 ± 0.208085 | 0.819192 ± 0.201728 | 2.44966 ± 0.896044 | 7.63E-14 | 1.66E-12 | A-B, A-D, B-C, B-D, C-D |
| PE 18:0_20:3 | 1.520971 ± 0.381222 | 1.383609 ± 0.483876 | 2.958534 ± 0.635872 | 2.783693 ± 1.028566 | 2.36E-05 | 5.55E-05 | A-D, B-C, B-D |
| PE 18:0_20:4 | 4.171813 ± 0.917084 | 6.174524 ± 2.053333 | 6.318792 ± 1.556932 | 9.031489 ± 3.416088 | 1.42E-06 | 4.27E-06 | A-B, A-D, B-C, B-D, C-D |
| PE 18:0_20:5 | 0.526632 ± 0.093485 | 0.519239 ± 0.058953 | 0.822854 ± 0.190183 | 0.986953 ± 0.388857 | 4.45E-05 | 9.68E-05 | A-D, B-D, C-D |
| PE 18:0_22:6 | 0.865504 ± 0.244604 | 0.761066 ± 0.101088 | 1.686073 ± 0.300172 | 1.455192 ± 0.515664 | 0.108033 | 0.116035 | |
| PE 18:1_18:1 | 0.875246 ± 0.202526 | 1.776092 ± 0.635529 | 1.123113 ± 0.25675 | 4.3796 ± 0.991115 | 1.16E-10 | 9.75E-10 | A-B, A-D, B-C, B-D, C-D |
| PE 18:1_18:2 | 0.29499 ± 0.062664 | 0.448663 ± 0.055782 | 0.437456 ± 0.091792 | 0.970372 ± 0.384407 | 9.75E-14 | 1.70E-12 | A-B, A-D, B-C, B-D, C-D |
| PE 18:1_20:3 | 0.671708 ± 0.173115 | 0.497559 ± 0.068161 | 1.349566 ± 0.275772 | 0.998653 ± 0.377188 | 0.000501 | 0.000779 | A-D, B-C, B-D |
| PE 18:1_20:4 | 1.707368 ± 0.322895 | 1.972093 ± 0.218194 | 2.915789 ± 0.650642 | 3.381174 ± 1.228661 | 0.000333 | 0.000537 | A-D, B-D, C-D |
| PE O-16:1_20:4 | 0.047078 ± 0.008939 | 0.089373 ± 0.012242 | 0.075724 ± 0.01796 | 0.165626 ± 0.063293 | 1.34E-10 | 9.75E-10 | A-B, A-D, B-C, B-D, C-D |
| PE O-18:2_22:6 | 0.014848 ± 0.002602 | 0.01363 ± 0.001713 | 0.024724 ± 0.005921 | 0.025076 ± 0.01173 | 0.525348 | 0.537709 | |
| PE O-20:1_20:4 | 0.012356 ± 0.006689 | 0.023651 ± 0.005084 | 0.020199 ± 0.005754 | 0.045005 ± 0.005781 | 0.087703 | 0.095377 | |
| PG 14:0_18:1 | 0.123646 ± 0.016983 | 0.148477 ± 0.016591 | 0.15334 ± 0.042284 | 0.197988 ± 0.083793 | 2.87E-10 | 1.79E-09 | A-B, A-C, A-D, B-C, C-D |
| PG 16:0_16:0 | 0.173697 ± 0.029941 | 0.256572 ± 0.029785 | 0.197137 ± 0.051127 | 0.283486 ± 0.119534 | 2.87E-10 | 1.79E-09 | A-B, A-C, A-D, B-C, C-D |
| PG 16:0_18:0 | 0.038269 ± 0.006858 | 0.033435 ± 0.008392 | 0.044398 ± 0.011372 | 0.046365 ± 0.026531 | 0.157269 | 0.166859 | |
| PG 16:0_18:1 | 2.009061 ± 0.356996 | 2.050039 ± 0.26873 | 2.777772 ± 0.635195 | 2.505211 ± 1.024155 | 0.001758 | 0.002352 | A-C, C-D |
| PG 16:1_18:1 | 0.090084 ± 0.01375 | 0.120918 ± 0.013159 | 0.129404 ± 0.03971 | 0.161439 ± 0.066794 | 2.76E-09 | 1.50E-08 | A-B, A-C, A-D, B-C, C-D |
| PG 18:0_18:1 | 0.317157 ± 0.055134 | 0.326905 ± 0.082943 | 0.507528 ± 0.110761 | 0.59295 ± 0.251332 | 0.00014 | 0.000276 | A-D, B-D, C-D |
| PG 18:1_18:1 | 0.37435 ± 0.065295 | 0.990221 ± 0.11346 | 0.623811 ± 0.181191 | 1.278914 ± 0.574 | 3.97E-14 | 1.15E-12 | A-B, A-D, B-C, C-D |
| PG 18:1_22:6 | 0.342159 ± 0.053801 | 0.338231 ± 0.034965 | 0.600706 ± 0.149581 | 0.53437 ± 0.217221 | 7.44E-05 | 0.000151 | A-D, B-D, C-D |
| PG 18:2_22:6 | 0.056976 ± 0.006406 | 0.063341 ± 0.005172 | 0.102698 ± 0.025736 | 0.09644 ± 0.045538 | 0.000558 | 0.000822 | A-D, B-D, C-D |
| PG 20:4_22:6 | 0.023694 ± 0.001648 | 0.046017 ± 0.00433 | 0.055302 ± 0.008897 | 0.069884 ± 0.04325 | 0.000206 | 0.000366 | A-B, A-D, C-D |
| PG 22:6_22:6 | 0.232372 ± 0.032234 | 0.318642 ± 0.039407 | 0.360999 ± 0.097315 | 0.273224 ± 0.109125 | 0.000542 | 0.000813 | A-B, B-C, B-D |
| PI 16:0_20:3 | 0.845469 ± 0.165183 | 0.368695 ± 0.039492 | 1.709622 ± 0.573347 | 0.48286 ± 0.164745 | 3.44E-09 | 1.72E-08 | A-B, A-D, B-C, C-D |
| PI 16:0_20:4 | 0.612027 ± 0.111067 | 0.626489 ± 0.062718 | 1.012616 ± 0.348081 | 0.528411 ± 0.200021 | 0.001629 | 0.002214 | A-D, B-D, C-D |
| PI 16:0_22:6 | 0.090377 ± 0.018083 | 0.060381 ± 0.009468 | 0.154759 ± 0.055933 | 0.020646 ± 0.014898 | 1.63E-07 | 5.91E-07 | A-D, B-D, C-D |
| PI 18:0_20:3 | 3.020445 ± 0.537145 | 1.738179 ± 0.437576 | 6.996068 ± 1.967753 | 4.657839 ± 1.704947 | 2.29E-06 | 6.04E-06 | A-B, A-C, B-C, B-D |

| | | | | | | | | | | | | | | | |
|---|---|---|---|---|---|---|---|---|---|---|---|---|---|---|---|
| PI 18:0_20:4 | 2.582931 | ± | 0.461282 | 4.430879 | ± | 0.413944 | 5.127251 | ± | 1.649738 | 6.872742 | ± | 2.625722 | 8.74E-07 | 2.78E-06 | A-B, A-D, B-C, C-D |
| PI 18:1_18:1 | 0.299421 | ± | 0.058191 | 0.177223 | ± | 0.013942 | 0.503345 | ± | 0.165384 | 0.17949 | ± | 0.064678 | 1.92E-06 | 5.39E-06 | A-B, A-D, B-C, C-D |
| PI 18:1_20:4 | 0.808338 | ± | 0.147084 | 0.703117 | ± | 0.063444 | 1.122485 | ± | 0.361779 | 0.61103 | ± | 0.242502 | 0.000408 | 0.000646 | A-D, B-D, C-D |
| PS 18:0_20:3 | 0.16006 | ± | 0.028269 | 0.153035 | ± | 0.035758 | 0.263548 | ± | 0.055793 | 0.261454 | ± | 0.104335 | 0.014104 | 0.017529 | B-D |
| PS 18:0_20:4 | 0.297623 | ± | 0.06174 | 0.472284 | ± | 0.12841 | 0.577834 | ± | 0.117136 | 0.55968 | ± | 0.225859 | 0.076302 | 0.084029 | |
| PS 18:0_22:6 | 0.314493 | ± | 0.063921 | 0.300834 | ± | 0.086607 | 0.610976 | ± | 0.107773 | 0.493976 | ± | 0.262302 | 0.030168 | 0.034995 | B-C |
| SM 18:0;2O/16:0 | 1.77337 | ± | 0.208257 | 1.561494 | ± | 0.18404 | 2.42408 | ± | 0.628146 | 2.03535 | ± | 0.951911 | 0.00152 | 0.002133 | A-B, A-C |
| SM 18:1;2O/16:0 | 4.425227 | ± | 0.631143 | 4.908999 | ± | 0.61355 | 7.267742 | ± | 1.977892 | 7.624466 | ± | 3.206822 | 8.95E-07 | 2.78E-06 | A-D, B-D, C-D |
| SM 18:1;2O/16:1 | 0.371911 | ± | 0.091693 | 0.344901 | ± | 0.055979 | 0.495695 | ± | 0.145635 | 0.520815 | ± | 0.253116 | 2.49E-05 | 5.70E-05 | A-C, B-D, C-D |
| SM 18:1;2O/22:0 | 0.269624 | ± | 0.041957 | 0.345885 | ± | 0.046816 | 0.4883 | ± | 0.107879 | 0.498147 | ± | 0.216674 | 4.40E-07 | 1.53E-06 | A-B, A-D, B-C, B-D, C-D |
| SM 18:1;2O/22:1 | 0.076626 | ± | 0.012148 | 0.092861 | ± | 0.011219 | 0.140809 | ± | 0.028859 | 0.144952 | ± | 0.066732 | 6.90E-08 | 2.61E-07 | A-B, A-D, B-D, C-D |
| SM 18:1;2O/24:0 | 0.200505 | ± | 0.0325 | 0.228628 | ± | 0.033245 | 0.304169 | ± | 0.072146 | 0.279176 | ± | 0.114491 | 0.000196 | 0.000362 | A-C, B-C, C-D |
| SM 18:1;2O/24:1 | 0.722814 | ± | 0.155531 | 0.814032 | ± | 0.116722 | 1.134801 | ± | 0.225446 | 1.14522 | ± | 0.508027 | 3.96E-05 | 8.83E-05 | A-D, B-C, B-D, C-D |
| TG 14:0_14:0_16:1 | 0.099045 | ± | 0.014926 | 0.066197 | ± | 0.009687 | 0.16253 | ± | 0.057252 | 0.052261 | ± | 0.035637 | 0.000218 | 0.000379 | A-D, B-D, C-D |
| TG 14:0_16:0_16:1 | 0.702679 | ± | 0.09379 | 0.531037 | ± | 0.051956 | 1.334441 | ± | 0.472077 | 0.310659 | ± | 0.141852 | 4.85E-07 | 1.62E-06 | A-D, B-D, C-D |
| TG 14:0_16:0_18:1 | 2.630419 | ± | 0.279419 | 2.20316 | ± | 0.286541 | 5.283448 | ± | 1.773131 | 1.253008 | ± | 0.520271 | 5.42E-06 | 1.39E-05 | A-D, B-D, C-D |
| TG 16:0_16:1_18:1 | 6.562757 | ± | 0.679601 | 5.492367 | ± | 0.695358 | 13.31198 | ± | 4.459034 | 3.395401 | ± | 1.414479 | 1.12E-05 | 2.70E-05 | A-D, B-D, C-D |
| TG 16:0_17:1_18:1 | 1.167072 | ± | 0.154659 | 1.247935 | ± | 0.193739 | 2.437687 | ± | 0.704879 | 0.882481 | ± | 0.348189 | 0.000324 | 0.000532 | A-D, B-D, C-D |
| TG 16:0_18:0_18:1 | 1.135396 | ± | 0.130378 | 1.65631 | ± | 0.714494 | 3.318677 | ± | 1.094591 | 0.984404 | ± | 0.36725 | 0.000166 | 0.000322 | A-C, B-D, C-D |
| TG 16:0_18:0_20:1 | 0.104149 | ± | 0.018521 | 0.18256 | ± | 0.075865 | 0.391797 | ± | 0.127038 | 0.11647 | ± | 0.042932 | 5.94E-05 | 0.000123 | A-B, A-C, B-D, C-D |
| TG 16:0_18:1_18:1 | 6.848388 | ± | 0.721262 | 7.183004 | ± | 1.16665 | 14.32494 | ± | 4.615405 | 5.646177 | ± | 2.2243 | 0.003401 | 0.004416 | B-D, C-D |
| TG 16:0_18:1_24:1 | 0.014814 | ± | 0.005416 | 0.025384 | ± | 0.013061 | 0.029785 | ± | 0.011051 | 0.006652 | ± | 0.002963 | 0.001933 | 0.002548 | B-D, C-D |
| TG 16:1_16:1_22:6 | 0.026209 | ± | 0.008753 | 0.049797 | ± | 0.018125 | 0.059965 | ± | 0.031508 | 0.050576 | ± | 0.035364 | 0.046393 | 0.052418 | |
| TG 16:1_18:1_18:1 | 5.648763 | ± | 0.725016 | 4.958885 | ± | 0.668202 | 11.39949 | ± | 3.442622 | 3.069841 | ± | 2.406419 | 0.023305 | 0.027399 | C-D |
| TG 16:1_18:1_18:2 | 1.014509 | ± | 0.133925 | 0.981242 | ± | 0.0965 | 1.867858 | ± | 0.643975 | 0.610484 | ± | 0.232262 | 4.68E-05 | 9.94E-05 | A-D, B-D, C-D |
| TG 16:1_18:1_20:4 | 0.244353 | ± | 0.050388 | 0.506071 | ± | 0.046891 | 0.415912 | ± | 0.187677 | 0.189935 | ± | 0.076085 | 2.24E-06 | 6.04E-06 | A-B, A-D, B-C, B-D |
| TG 17:0_18:0_18:1 | 0.062203 | ± | 0.01487 | 0.088671 | ± | 0.048655 | 0.183043 | ± | 0.054604 | 0.050906 | ± | 0.013307 | 0.018446 | 0.021983 | C-D |
| TG 17:1_18:1_18:2 | 0.195433 | ± | 0.060234 | 0.27334 | ± | 0.074471 | 0.382208 | ± | 0.144178 | 0.161028 | ± | 0.065237 | 0.001624 | 0.002214 | B-D |
| TG 18:1_18:1_18:1 | 3.938707 | ± | 0.477433 | 4.189722 | ± | 0.711902 | 8.347327 | ± | 2.488469 | 2.269194 | ± | 2.780761 | 0.001329 | 0.001895 | A-D, B-D, C-D |
| TG 18:1_18:1_18:2 | 1.78212 | ± | 0.230518 | 2.001966 | ± | 0.307897 | 3.307012 | ± | 1.507455 | 1.556386 | ± | 1.210426 | 0.014404 | 0.01765 | B-D |
| TG 18:1_18:1_20:1 | 0.472209 | ± | 0.081565 | 0.56282 | ± | 0.259019 | 1.070228 | ± | 0.327624 | 0.467566 | ± | 0.166363 | 0.035153 | 0.040241 | C-D |

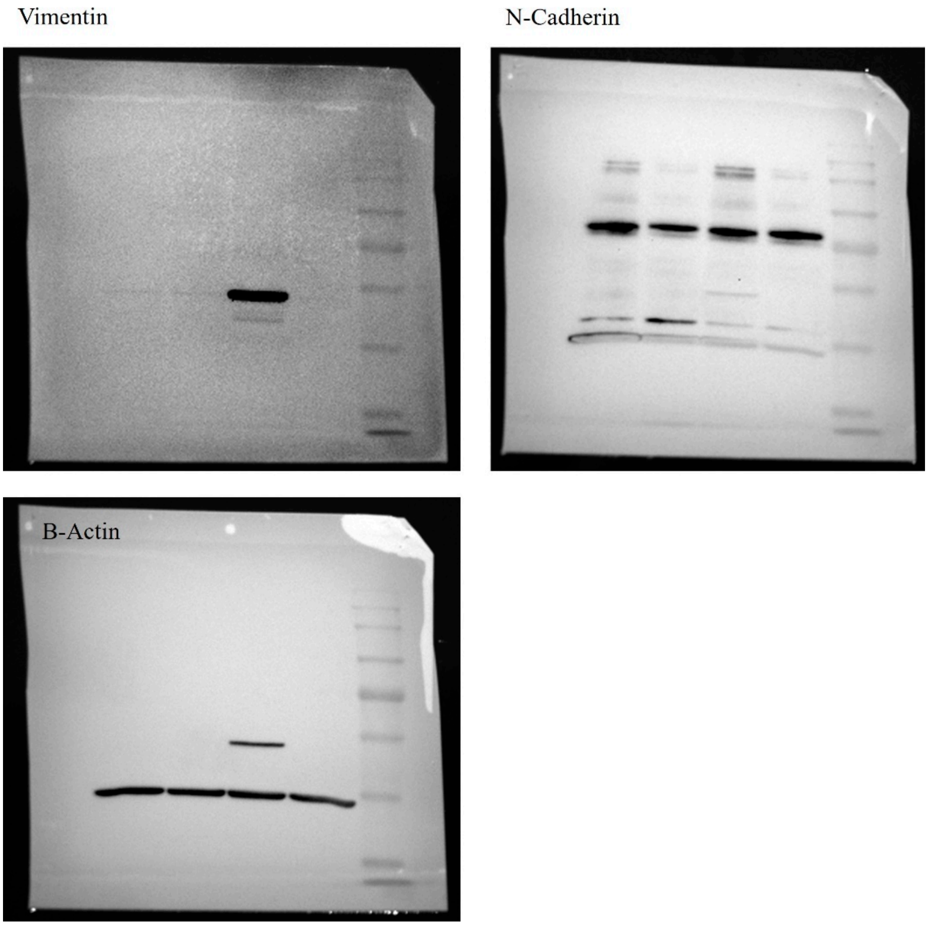


**Figure S4.** Full, uncropped Western blot images corresponding to Figure 3c.

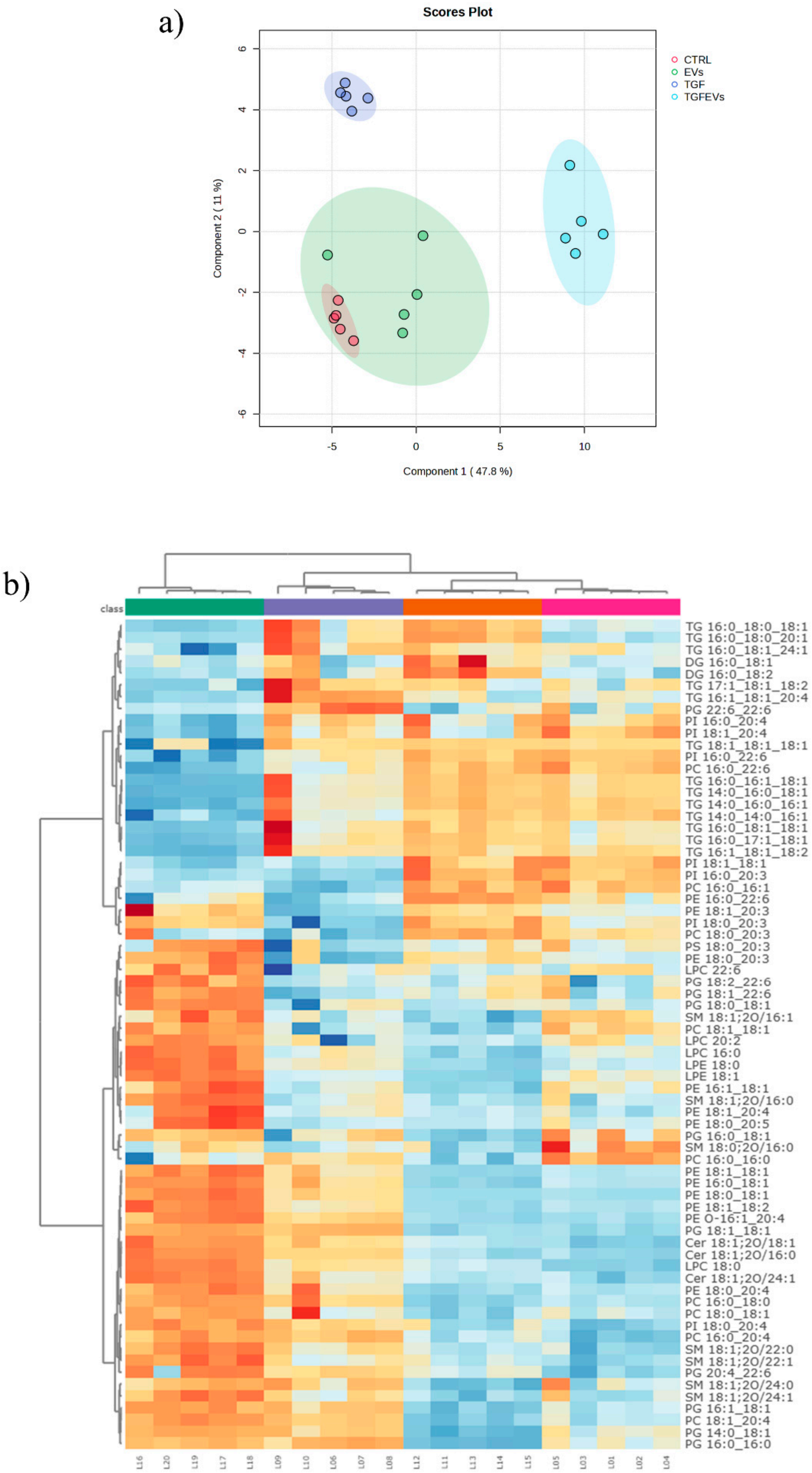


**Figure S5.** Lipidomic Analysis of caper EVs treatment of Huh7-induced EMT. Partial Least Squares Discriminant Analysis (PLS-DA) score plot (b), hierarchical clustering Heatmap (b). In panel b, the color scale represents the relative lipid abundance (log2 fold change), ranging from blue (lower expression) to red (higher expression). In panel C, the heatmap shows lipid expression levels across groups, with colors from blue (low)

to red (high) expression. Data were normalized by median centering, log-transformed, and autoscaled before multivariate analysis using LipidOne 2.3.

**Table S3.** Enriched metabolic pathways identified from integrative metabolomic–lipidomic correlation analysis. Pathways are ranked according to statistical significance and topology-based impact score; raw p-values, Holm correction, and FDR-adjusted values are reported.

| | | | | | | |
|---|---|---|---|---|---|---|
| Glycine, serine and threonine metabolism | 4/33 | 9.2657E-6 | 5.0331 | 3.0577E-4 | 3.0577E-4 | 0.26608 |
| Pyrimidine metabolism | 3/39 | 3.2429E-5 | 4.4891 | 0.0010377 | 5.3507E-4 | 0.01345 |
| Arginine and proline metabolism | 4/36 | 9.5679E-5 | 4.0192 | 0.0029661 | 0.0010525 | 0.16628 |
| Nicotinate and nicotinamide metabolism | 1/15 | 3.0693E-4 | 3.513 | 0.009208 | 0.0025322 | 0.13816 |
| Ether lipid metabolism | 1/20 | 0.0010369 | 2.9843 | 0.030069 | 0.0068432 | 0.0 |
| Glycerophospholipid metabolism | 5/36 | 0.001334 | 2.8748 | 0.037352 | 0.0073369 | 0.20972 |
| Lysine degradation | 2/30 | 0.0016174 | 2.7912 | 0.043669 | 0.0076247 | 0.0 |
| Purine metabolism | 5/70 | 0.0091983 | 2.0363 | 0.23916 | 0.037943 | 0.09843 |
